# Synergistic Ultramicropore-Confined and Electronic-State Modulation Strategies in Sustainable Lignin-Derived Hard Carbon for Robust Sodium-Ion Batteries

**DOI:** 10.34133/research.1039

**Published:** 2026-01-15

**Authors:** Yuzhong Xie, Yuqing Wang, Yusuke Yamauchi, Minjun Kim, Fang Yuan, Yuhang He, Yiqiang Wu, Caichao Wan

**Affiliations:** ^1^ College of Materials and Energy, Central South University of Forestry and Technology, Changsha 410004, P. R. China.; ^2^ Australian Institute for Bioengineering and Nanotechnology (AIBN), The University of Queensland, Brisbane, QLD 4072, Australia.; ^3^ Department of Materials Process Engineering, Graduate School of Engineering, Nagoya University, Nagoya 464-8603, Japan.

## Abstract

The performance of hard carbon anodes in sodium-ion batteries is restricted by competing mechanisms: excessive surfaces cause irreversible reactions lowering the initial coulombic efficiency, while insufficient active sites limit capacity. To mitigate this trade-off effect, a synergistic strategy of ultramicropore confinement and electronic-state modulation in lignin-derived hard carbon was created. Using sodium lignosulfonate, a common sulfonated polymer in paper-making waste, we developed N/S-codoped hard carbon microspheres (N-S@HDM) via preoxidation-induced cross-linking and optimized pyrolysis. Preoxidation inhibits graphitic alignment, creating an expanded interlayer spacing and a closed-pore-dominated structure (94.27% at 1,300 °C). This interconnected network (*f*_a_ = 0.85) enables ultramicropore confinement, thus shortening diffusion paths, boosting kinetics, and providing ample Na^+^ storage sites to reduce interfacial decomposition. Concurrent N/S doping optimizes electronic states by enhancing electron delocalization, lowers charge-transfer resistance, and generates high-density adsorption sites. The optimized N-S@HDM-1300 achieves an ultrahigh initial coulombic efficiency of 90.6% and a reversible capacity of 401.5 mAh g^−1^ (0.03 A g^−1^), with exceptional cyclability (95.0% retention after 500 cycles). This study pioneers a dual-regulation paradigm for biomass-derived carbon materials, coupling pore engineering and electronic optimization to advance sodium-ion battery anode design.

## Introduction

Driven by the rapid expansion of renewable energy installations and the ongoing global energy transition, sodium-ion batteries (SIBs) emerge as a key candidate for next-generation electrochemical energy storage. The advantages of SIBs include the abundancy of sodium resources (2.75% Na in the earth’s crust as compared to 0.006% Li), high cost-effectiveness, and electrochemical charging/discharging mechanisms similar to those of lithium-ion batteries (rocking-chair-type redox reactions) [1–3]. Unlike Li-ion batteries that commonly use graphite anodes, SIBs perform poorly in energy storage as graphite is employed as the anode. The main challenges stem from 2 factors: the larger ionic radius of Na^+^ (1.02 Å vs. 0.76 Å for Li^+^, Shannon crystal radii) and the thermodynamic instability of sodium–graphite intercalation compounds [4,5]. These factors consequently result in rapid capacity decay and insufficient cycle life for SIBs. Among various nongraphitic carbon anode materials, hard carbon, characterized by its highly disordered structure and expanded interlayer spacing (above 0.34 nm), stands out as a promising SIB anode material due to its high sodium storage capacity, low working voltage (below 0.3 V vs. Na^+^/Na), and long cycle life. Notably, the 3-stage sodium storage mechanism in hard carbon—“adsorption–intercalation–pore filling”—provides a new theoretical framework for overcoming the limitations of traditional carbon materials in sodium storage [6,7].

As a promising anode material for SIBs, hard carbon still faces 2 marked scientific challenges as follows: (a) the initial coulombic efficiency (ICE) generally remains low (50% to 80%) and (b) the reversible capacity is distinctly lower (typically below 300 mAh g^−1^). The research community has explored these issues, with precursor optimization and carbonization condition regulation being early strategies. For instance, Hong et al. [8] used phosphoric acid-activated pomelo peel as a precursor to prepare 3-dimensional (3D) porous hard carbon through pyrolysis at 700 °C. This material exhibited a high specific surface area of 1,272 m^2^ g^−1^, with mesopores (2 to 30 nm) dominating its pore size distribution. At a current density of 50 mA g^−1^, it delivered initial charge/discharge capacities of 1,149.7 and 314.5 mAh g^−1^, respectively, but achieved an ICE of only 27%. This indicates that there is no clear correlation between the surface area of anode materials and the ICE of SIBs. Although anode materials with a high specific surface area and abundant nanopores can deliver a high initial charge capacity, they often experience serious capacity losses (~70%) because of excessive electrode–electrolyte interfaces, where irreversible electrolyte decomposition, thick solid–electrolyte interphase (SEI) formation, and co-intercalation side reactions (e.g., with ester solvents: ethylene carbonate/dimethyl carbonate) occur. To address this, researchers have proposed the engineering of closed ultramicropores (below 0.7 nm) while reducing the volume of open mesopores [9,10]. Their narrow channels enable molecular-sieving effects that allow permeation of desolvated Na^+^ while blocking solvent molecules (e.g., ethylene carbonate with a kinetic diameter of 0.62 nm), thus suppressing excessive SEI growth. However, sacrificing open pores inevitably results in a reduction of surface adsorption capacity.

Heteroatom doping (e.g., boron, nitrogen, phosphorus, and sulfur) is another critical strategy for modulating the sodium storage abilities of hard carbon materials [11–13]. The introduced guest heteroatoms in the intrinsic carbon structure effectively generate active sites and structural defects, expand interlayer spacings, and optimize electron transport networks, thereby enhancing reversible capacity [14,15]. A typical example is nitrogen-doped 3D mesoporous carbon nanosheets, reported by Huang et al. [16], whose unique pore architecture and nitrogen-active sites achieves a high reversible capacity of 462 mAh g^−1^ at 0.1 A g^−1^. However, such materials with a high mesoporous content still suffer from a markedly reduced ICE because of excessive electrolyte–electrode interfacial contact. Although targeted modification strategies (e.g., pore size regulation and electronic-state modulation) hold a potential to improve individual performance metrics, resolving the trade-off effect between ICE and capacity remains a challenge [17,18]. Notably, among highly prioritized sustainable biomass-derived hard carbon, reports simultaneously achieving a high ICE (above 80%) and a substantial capacity (above 350 mAh g^−1^) remain exceptionally scarce.

Herein, we present sodium lignosulfonate (SLS), a byproduct of the paper-making industry, as a carbon precursor for a synergistic “ultramicropore confinement–electronic-state modulation” strategy to create nitrogen/sulfur-codoped hard carbon microspheres (N-S@HDM) featuring an expanded layer spacing and abundant closed ultramicropores. The crucial innovation lies in the preoxidation at 300 °C in air to induce molecular cross-linking and introduce oxygen-containing groups, therefore effectively suppressing the directional alignment of graphitic microcrystals during subsequent high-temperature pyrolysis (1,100 to 1,500 °C) and generating an expanded interlayer spacing structure and increasing the proportion of closed pores. As the pyrolysis temperature is optimized at 1,300 °C, the carbon material shows an optimal closed pore structure (closed pore ratio of up to 94.27%). At a relatively lower temperature of 1,100 °C, residual O/S groups hinder the ordering of graphitic carbon stacks, resulting in an open pore-dominant structure. In contrast, at a relatively higher temperature of 1,500 °C, excessive graphitization causes pore collapse. The synergistic effect from the codoped sulfur and nitrogen markedly enhances the electron transport network and creates high-density sodium adsorption sites. Benefiting from this multidimensional regulation, N-S@HDM-1300 displays exceptional sodium storage performance with a reversible capacity of 401.5 mAh g^−1^ (at 0.03 A g^−1^), an ICE of 90.6%, and a capacity retention of 95.0% after 500 cycles. When assembled into a full cell, the device delivers an energy density of 215.5 Wh kg^−1^ at a power density of 36.0 W kg^−1^, along with excellent rate capability.

## Results

### Preoxidation-induced covalent cross-linking and microstructural changes

SLS was selected as the precursor of N-S@HDM due to following advantages: (a) its intrinsic sulfur groups act as a self-doping source for defect engineering and electronic modulation; (b) its cross-linkable structure enables preoxidation to create a carbon matrix with expanded interlayers and closed pores, key to reconciling capacity and ICE; and (c) SLS is abundant and low-cost, ensuring sustainability and scalability.

**Fig. 1.**
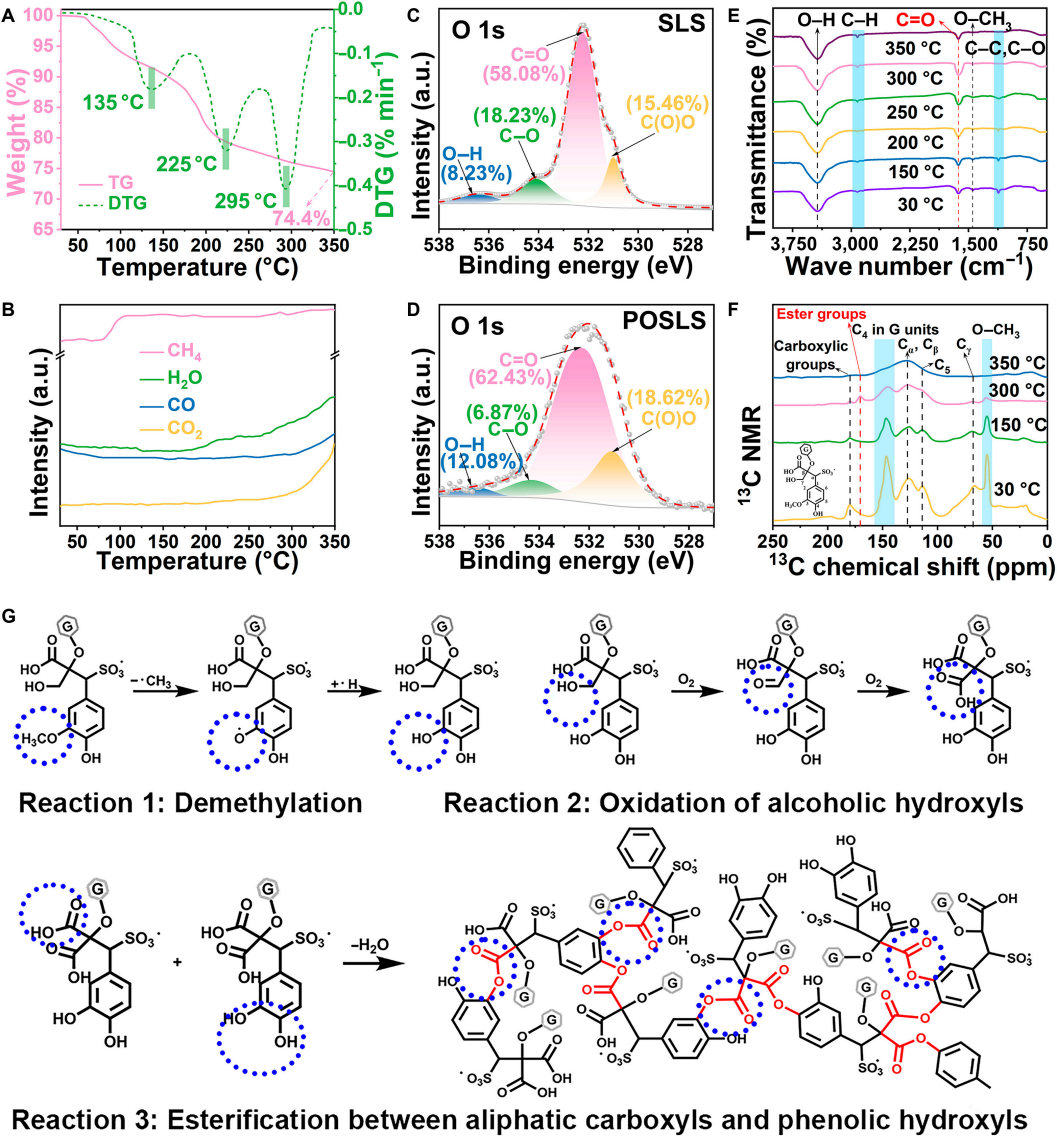
Preoxidation-induced covalent cross-linking of sodium lignosulfonate (SLS). (A and B) Thermogravimetric–mass spectrometry (TG–MS) analysis revealing the thermal decomposition behavior. (C and D) X-ray photoelectron spectroscopy (XPS) O 1s spectral deconvolution for (C) pristine SLS and (D) preoxidized SLS (POSLS). (E) Fourier transform infrared (FTIR) and (F) ^13^C nuclear magnetic resonance (NMR) spectra of SLS at different temperatures. (G) Proposed preoxidation mechanism highlighting covalent cross-linking through hydroxyl and carbonyl functionalities. DTG, derivative TG.

The preoxidation mechanism of SLS was quantitatively and qualitatively revealed by an integrated analysis of thermogravimetric–mass spectrometry (TG–MS), x-ray photoelectron spectroscopy (XPS), Fourier transform infrared (FTIR) spectroscopy, and ^13^C nuclear magnetic resonance (NMR) spectroscopy. In the preoxidation step, the structure of SLS undergoes stepwise transformations:

1. 30 to 180 °C: Release of adsorbed and bound water and demethylation
  In this region, the TG–derivative TG reveals a weight loss of ~13.2% (Fig. 1A), mainly associated with the release of adsorbed and bound water, while MS simultaneously detects the evolution of CH_4_ (Fig. 1B), indicating the cleavage of methoxy groups. In the O 1s XPS spectra (Fig. 1C and D and Table S1), the fraction of O2 (C–O, 534.1 eV) decreases from 18.23% to 6.87%, accompanied by an increase in O1 (O–H, 536.6 eV) from 8.23% to 12.08%, suggesting demethylation of methoxy substituents into hydroxyl groups [19]. Consistently, FTIR (Fig. 1E) shows attenuation of the O–CH_3_ deformation vibration (1,470 cm^−1^) and strengthening of the O–H stretching vibration (3,430 cm^−1^), while ^13^C NMR (Fig. 1F) exhibits a gradual decrease in the O–CH_3_ resonance at 53 ppm. Collectively, these results confirm that preoxidation promotes demethylation at low temperatures, thereby generating reactive hydroxyl sites for subsequent esterification.
2. 180 to 325 °C: Esterification and cross-linking
  In the intermediate-temperature range, TG–MS (Fig. 1A and B) detects intensified release of H_2_O, indicating decomposition of unstable functionalities such as aliphatic alcohols, aromatic alcohols, and aliphatic acids. Correspondingly, the O 1s XPS spectra show an increase in O3 (C=O, 532.5 eV) from 58.08% to 62.43% and in O4 (C(O)O, 530.9 eV) from 15.46% to 18.62% (Fig. 1C and D), proving the enrichment of carbonyl and ester groups. FTIR analysis (Fig. 1E) reveals that the C=O stretching vibration (1,630 cm^−1^) reaches its maximum intensity at 300 °C, while ^13^C NMR (Fig. 1F) demonstrates a pronounced enhancement of the ester resonance at 172 ppm. These observations collectively suggest that preoxidation facilitates esterification reactions between carboxyl and phenolic hydroxyl groups, strengthening interchain cross-linking and improving the structural stability of the carbon precursor.
3. Above 325 °C: Aromatization and structural stabilization
  Above 325 °C, TG–MS (Fig. 1A and B) continues to show mass loss accompanied by intensified CO_2_, CO, and H_2_O signals in MS. Meanwhile, the C=O band in FTIR (Fig. 1E) diminishes, and the ester resonance in ^13^C NMR disappears (Fig. 1F), leaving dominant aromatic carbon signals at 126 ppm (C_α_/C_β_). These results indicate that ester groups undergo further condensation and transform into aromatic C–C structures. Thus, at elevated temperatures, preoxidation drives aromatization, which ultimately stabilizes the lignin framework and prepares it for the formation of a disordered carbon skeleton.

In summary, preoxidation enables the sequential structural evolution of lignin: release of adsorbed and bound water and demethylation at 30 to 180 °C, esterification and cross-linking at 180 to 325 °C, and aromatization above 325 °C. The overall function of preoxidation lies in tailoring the chemical structure of the SLS precursor to promote interchain cross-linking and structural stability, thus effectively suppressing graphitic microcrystal alignment during the following high-temperature carbonization [20], reducing parallel carbon layer stacking [21], and thus promoting hard carbon formation.

Based on these above findings, Fig. 1G illustrates a possible reaction mechanism during SLS preoxidation, involving 3 key processes: (a) demethylation of SLS to generate alcoholic hydroxyl groups, (b) oxidation of hydroxyl groups to aliphatic carboxyl structures, and (c) cross-linking between the carboxyl and hydroxyl groups. This preoxidation-induced covalent cross-linking facilitates the formation of a compact and stable 3D molecular network, effectively mitigating the severe decomposition of SLS during the subsequent pyrolysis. The resulting structure is anticipated to not only increase carbon layer spacing but also reduce open porosity while concurrently forming a multitude of closed pores within the hard carbon [22].

To evaluate the effect of preoxidation on the structure of pyrolyzed carbon, x-ray diffraction (XRD), transmission electron microscopy (TEM), and Raman analyses were conducted on hard carbon microspheres (HDMs) derived from both pristine SLS and preoxidized SLS (POSLS) (labeled as HDM-NPU and HDM-NU, respectively). From Fig. S1 (Supplementary Materials), the XRD pattern of HDM-NPU displays 2 broad peaks near 25° and 46°, corresponding to the (002) and (100) planes of amorphous graphite, respectively [23]. Compared to that of HDM-NPU, the peak of the (002) plane shifts to a lower 2*θ* angle for HDM-NU, reflecting an expanded interlayer spacing [24]. Based on the Bragg equation [25], the interlayer distance between (002) planes (*d*_002_) increases from 0.358 nm (HDM-NU) to 0.372 nm (HDM-NPU), consistent with the high-resolution TEM observations (Fig. S2a and b). This indicates that preoxidation not only induces molecular cross-linking but also enlarges the carbon layer spacing, beneficial for rapid Na^+^ de/intercalation [26]. Raman spectra (Fig. S3) show an increased D/G band intensity ratio (*I*_D_/*I*_G_) from 0.98 (HDM-NPU) to 1.13 (HDM-NU), further suggesting the enhanced structural disorder caused by the preoxidation. HDM-NU has greater closed pore surface area (195.35 m^2^ g^−1^) and proportion (96.25%), respectively, compared to HDM-NPU (124.01 m^2^ g^−1^ and 19.37%) (Figs. S4 and S5 and Table S2). This structural regulation is expected to exhibit beneficial effects for sodium storage applications.

### Pyrolysis-driven N–S doping and pore architecture evolution

**Fig. 2.**
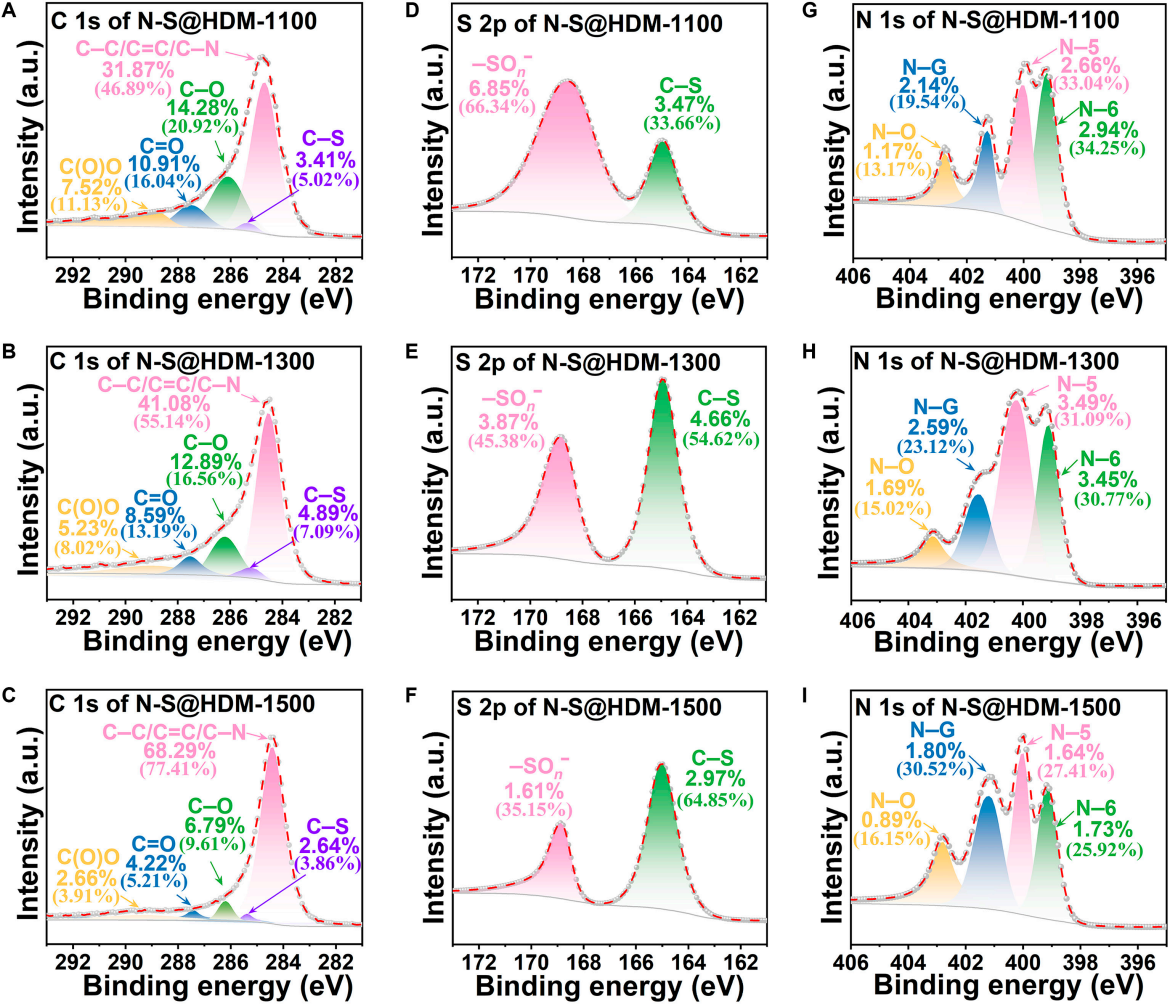
Temperature-driven modulation of N/S synergistic doping in N/S-codoped hard carbon microspheres (N-S@HDM). XPS spectral deconvolution of the (A to C) C 1s regions, (D to F) S 2p regions, and (G to I) N 1s regions of N-S@HDM synthesized at 1,100, 1,300, and 1,500 °C. Note: The percentage values within parentheses indicate relative content, while those outside parentheses represent actual content.

After the preoxidation, POSLS was mixed with urea and then subjected to high-temperature pyrolysis, to obtain N/S-codoped carbon materials. The pyrolysis-driven N–S doping mechanism was investigated using XPS analysis. Figure S6 exhibits the XPS survey spectra of all N-S@HDM samples prepared at 1,100, 1,300, and 1,500 °C, where characteristic peaks of the C, O, N, and S elements are clearly identified. The C 1s spectra (Fig. 2A to C) are deconvoluted into 5 characteristic peaks: C1 (C(O)O), C2 (C=O), C3 (C–O), C4 (C–C/C=C/C–N), and C5 (C–S) (specific proportions are listed in Table S3) [27]. When the pyrolysis temperature increases from 1,100 to 1,500 °C, both the actual content (values outside parentheses) and relative content (values in parentheses) of oxygen-containing groups (C1, C2, and C3) decrease markedly, while the proportion of C4 rises. This indicates that the high temperature promotes the decomposition of oxygen-containing groups and enhance the formation of graphitic structures. Such structural evolution aligns well with the conductivity changes (4-probe tests prove that the electrical conductivity increases from 175.14 ± 1.90 to 354.70 ± 3.61 S m^−1^ as the pyrolysis temperature rises from 1,100 to 1,500 °C; Fig. S7, Supplementary Materials), proving the key role of a high C4 content in the conductivity improvement. The persistent presence of C5 (C–S bonds) demonstrates successful sulfur self-doping into the carbon skeleton.

The chemical states of sulfur were further studied through S 2p spectra (Fig. 2D to F). Spectral deconvolution reveals 2 characteristic peaks: S1 (–SO*_n_*^−^) and S2 (C–S). With increasing temperature, the relative content of S1 decreases from 66.34% to 35.15%, while the relative content of S2 increases from 33.66% to 64.85%, indicating that high temperatures facilitate the conversion of sulfonic groups (–SO*_n_*^−^) to C–S bonds. Notably, the actual content of S2 peaks at 4.66% at 1,300 °C but declines to 2.97% at 1,500 °C, revealing the excessive thermal decomposition of sulfur species at the extreme temperature (1,500 °C). The performance enhancement from sulfur self-doping mainly stems from the following: (a) the comparable electronegativities of the S element (2.58) and carbon element (2.55) promote conjugated structure formation, enhancing electron delocalization and reducing charge-transfer resistance [28]; (b) the larger atomic radius of S (0.104 nm vs. C: 0.077 nm) expands the carbon interlayer spacing, thereby lowering the Na^+^ intercalation energy barrier [29]; and (c) sulfur-induced topological defects (e.g., pentagon/heptagon rings) and vacancies create additional sodium storage active sites [30].

The effect of urea copyrolysis on the nitrogen doping behavior was systematically investigated through N 1s XPS spectral analysis of N-S@HDM (Fig. 2G to I). Spectral deconvolution reveals 4 characteristic peaks: N1 (N–O, oxidized nitrogen), N2 (N–G, graphitic nitrogen), N3 (N–5, pyrrolic nitrogen), and N4 (N–6, pyridinic nitrogen) [31]. With increasing pyrolysis temperature, the relative contents of N–5 (pyrrolic N) and N–6 (pyridinic N) gradually decrease, while the relative contents of N–O and N–G increase markedly, indicating that elevated temperatures promote the conversion of pyridinic/pyrrolic nitrogen into thermodynamically more stable oxidized and graphitic nitrogen. Notably, under moderate-temperature conditions (1,300 °C), the total actual content of the 4 nitrogen components reaches its peak, confirming this temperature as the optimal range for achieving efficient nitrogen doping. Regarding the functional mechanisms, the pentagonal ring structure of N–5 enhances the local electronegativity through an unsaturated electronic environment, inducing electron density redistribution in adjacent carbon atoms and thereby improving the material’s electrical conductivity (Fig. S8) [32]. Meanwhile, N–G is embedded in the graphitic carbon layer via planar tricoordination, forming stable covalent bonds, where the lone electron pairs of nitrogen directly participate in the π-conjugation system of the carbon skeleton, further enhancing electron transport capability [32]. In summary, the codoping of S and N synergistically regulates both the chemical bonding (e.g., C–S/C–N bonds) and electronic structure (e.g., charge delocalization) of the carbon framework, markedly optimizing charge transfer and storage performance, which provides a critical theoretical foundation for the design of SIB electrodes.

**Fig. 3.**
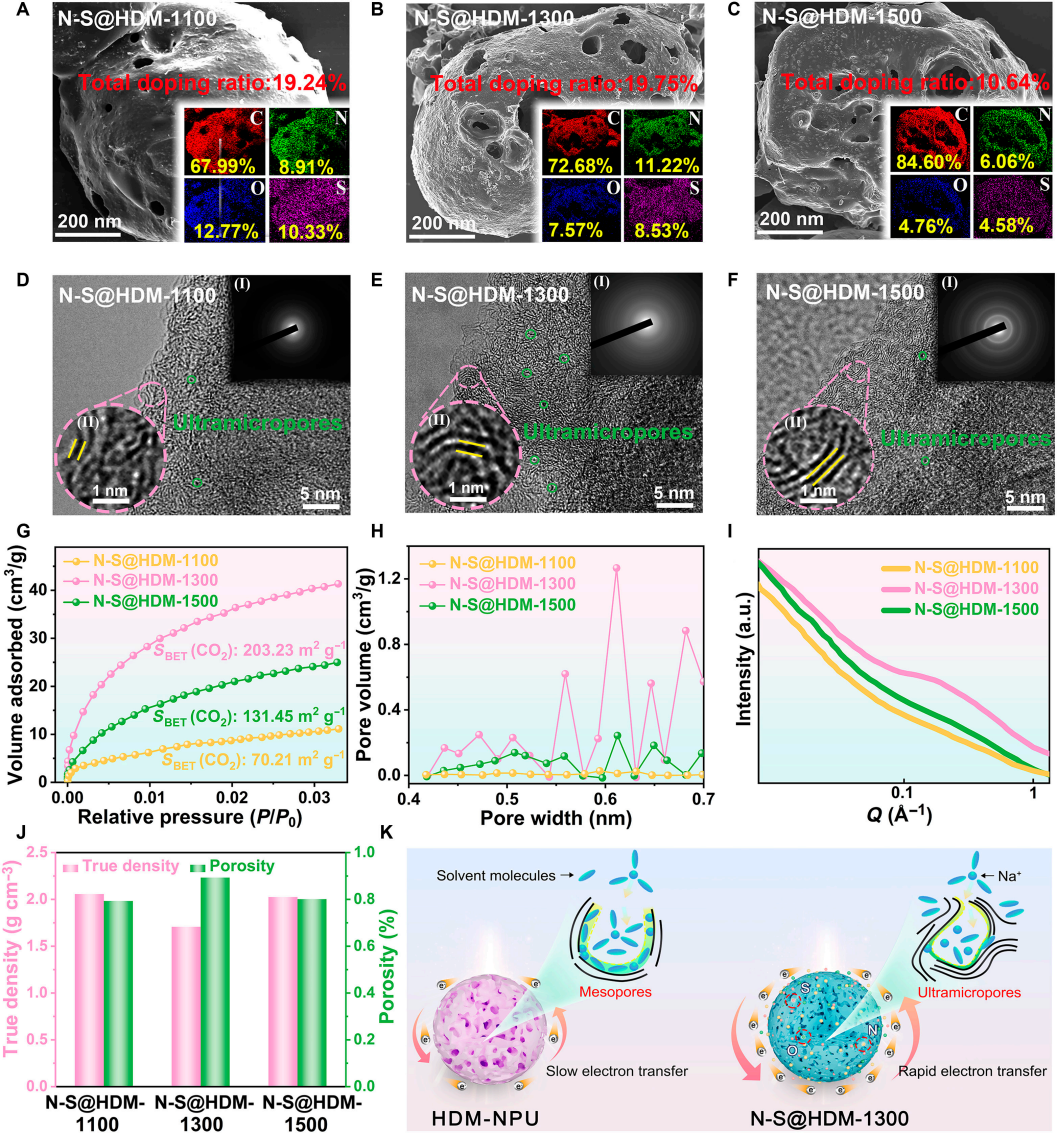
Temperature-driven modulation of pore architecture in N-S@HDM. (A to C) Scanning electron microscopy (SEM) images and corresponding energy-dispersive x-ray spectroscopy (EDS) mapping patterns. (D to F) High-resolution transmission electron microscopy (HRTEM) images and associated selected-area electron diffraction (SAED) patterns. (G) CO_2_ absorption–desorption isotherms. (H) Pore size distribution profiles. (I) Small-angle x-ray scattering (SAXS) patterns. (J) True density and porosity. (K) Schematic illustration highlighting the advantages of “the synergistic ultramicropore-confined and electronic-state modulation strategies” on Na^+^ intercalation/deintercalation processes. HDM-NPU, SLS-derived hard carbon microspheres prepared without preoxidation and urea.

Scanning electron microscopy (SEM) was further employed to study the modulation mechanisms of pyrolysis temperature on the pore architecture and surface electronic states of N-S@HDM. As shown in Fig. 3A to C, the decomposition of SLS becomes more intense with increasing pyrolysis temperature, leading to gradually roughened surface structures of the obtained N-S@HDM. Figure S9 reveals that the electrode crafted from N-S@HDM-1300 has superior tensile strength, with notable enhancements of 13.68% and 9.17% compared to those fabricated at 1,100 and 1,500 °C, respectively. This observation underscores the contribution of the augmented surface roughness of hard carbon to the heightened cohesion of the electrode. Additionally, the elemental mapping images shown in the insets of Fig. 3A to C reveal the uniform doping of the N and S elements throughout the carbon network, with doping ratio data (Table S4) consistent with the aforementioned XPS analysis. Moreover, the total N–S doping ratio of N-S@HDM-1300 reaches 19.75%, surpassing those of N-S@HDM-1100 (19.24%) and N-S@HDM-1500 (10.64%). The incorporation of the N and S elements is anticipated to optimize the electronic structure, facilitating enhanced charge delocalization and introducing defect sites, thus providing additional electrochemically active sites [33]. These theoretical aspects will be elaborated in the subsequent density functional theory (DFT) calculation.

Figure 3D to F show the high-resolution TEM images of N-S@HDM prepared at various pyrolysis temperatures. At 1,100 °C (Fig. 3D), the material exhibits a highly disordered carbon layer structure with a larger interlayer spacing (*d*_002_ = 0.399 nm, calculated through the Bragg equation [24] according to the XRD data in Fig. S10 and Table S5) and broad, diffuse selected-area electron diffraction (SAED) rings. This morphology is ascribed to incomplete deoxygenation and desulfurization at this temperature, resulting in poor carbon layer planarity and insufficient cross-linking. Upon raising the temperature to 1,300 °C (Fig. 3E), localized graphitic microcrystals (*D* = 1.01 nm, calculated via the Scherrer equation [34] based on the XRD data) are formed, accompanied by a reduced interlayer spacing (*d*_002_ = 0.389 nm). The increased structural curvature (as indicated in the green circles in Fig. 3E) and the presence of topological defects (e.g., pentagon/heptagon ring distortions) create a morphology highly conducive to the formation of subnanometer pores (like ultramicropores), as illustrated in the schematic (Fig. S11) [35]. This structural evolution corresponds to a marked development of the pore architecture, the quantitative analysis of which is discussed in the following section. As the temperature further rises to 1,500 °C (Fig. 3F), the graphitic microcrystals grow larger (*D* = 1.9 nm) and the interlayer spacing further narrows (*d*_002_ = 0.375 nm). The enhanced carbon layer planarization and coalescence of graphitic domains lead to a reduction in the nanostructural features that host subnanometer pores.

In addition, notably, N-S@HDM-1500 shows sharper and more continuous diffraction rings. The underlying mechanism involves enhanced carbon layer planarization at the high temperature, resulting in pore collapse due to graphitic microcrystal coalescence and a gradual transition toward ordered carbon stacking [36]. These results demonstrate that the pyrolysis at 1,300 °C achieves a balanced structure of short-range order (graphitic microcrystals) and long-range disorder (curved carbon layers), while the excessively high temperature (1,500 °C) drives the irreversible loss of closed pores by transforming hard carbon into graphite-like structures. The monotonic decline in the *I*_D_/*I*_G_ ratio of the Raman spectra (Fig. S12, Supplementary Materials) of N-S@HDM-1100, N-S@HDM-1300, and N-S@HDM-1500 with the increasing pyrolysis temperature (from 1.22 to 0.97) provides further evidence.

To study the regulation mechanism of pyrolysis temperature on the pore structure of hard carbon, combined N_2_ (77 K) and CO_2_ (273 K) adsorption analyses were employed. According to the International Union of Pure and Applied Chemistry pore classification criteria [37], N_2_ adsorption is appropriate for characterizing mesopores (between 2 and 50 nm) and supermicropores (between 0.7 and 2 nm), while CO_2_ adsorption precisely detects ultramicropores (less than 0.7 nm). N_2_ adsorption results (Fig. S13) suggest that as the pyrolysis temperature increases from 1,100 to 1,300 and 1,500 °C, the specific surface area of N-S@HDM sharply decreases from 603.45 to 21.78 and 12.35 m^2^ g^−^1 (Table S6), corresponding to decreases of 96.4% and 98.0%, respectively, revealing the dramatic collapse of mesopores and larger micropores (above 0.7 nm). The reduction of such pores inhibits deep electrolyte penetration and minimizes active surface area exposure, thus alleviating the excessive SEI film growth during Na^+^ intercalation/deintercalation, which structurally ensures a high ICE.

CO_2_ adsorption results (Fig. 3G and H) indicate that the ultramicropore specific surface area of N-S@HDM-1300 reaches 203.23 m^2^ g^−1^, representing 189.5% and 54.6% improvements compared with those of N-S@HDM-1100 (70.21 m^2^ g^−1^) and N-S@HDM-1500 (131.45 m^2^ g^−1^), respectively. This demonstrates that moderate pyrolysis (1,300 °C) facilitates the formation and retention of subnanometer closed pores. Such closed pores act as “Na^+^ storage cabins”, enhancing the plateau-region capacity through adsorption-filling mechanisms. Small-angle x-ray scattering (SAXS) was further used to quantify pore connectivity (Fig. 3I). In the structural characterization of porous materials, the amphiphilic factor (*f*_a_), a critical order parameter derived from SAXS analysis according to the Teubner–Strey model [38], quantifies the competitive ordering within the material’s internal architecture. As *f*_a_ approaches 0, the system has isolated closed pores or short-range disordered structures. Conversely, as *f*_a_ approaches 1, it signifies the formation of 3D interconnected pore channels, a structural configuration characterized by short-range periodic order and long-range disorder. N-S@HDM-1300 exhibits a markedly higher *f*_a_ value of 0.85 compared to N-S@HDM-1100 (0.51) and N-S@HDM-1500 (0.69), as summarized in Table S6. This enhancement in *f*_a_ suggests improved pore channel connectivity and reduced Na^+^ diffusion energy barriers, which, in conjunction with the high ultramicropore specific surface area, optimize the rate performance.

Helium-pycnometry-based true density and porosity analyses (Fig. 3J and Table S7) suggest that N-S@HDM-1300 exhibits optimized closed pore structural features: its true density is 1.72 g cm^−3^, corresponding to a total porosity of 89.25%, which is 13.0% and 12.9% higher than those of N-S@HDM-1100 (78.97%) and N-S@HDM-1500 (79.06%), respectively. This further verifies the abundant closed ultramicropores in N-S@HDM-1300. The integration of these tailored structural and compositional features establishes the foundation for a synergistic strategy in hard carbon design. Specifically, (a) the ultramicropore confinement is demonstrated by the high fraction of closed pores (94.27%, Table S6) and interconnected network (*f*_a_ = 0.85), which is anticipated to provide abundant, confined storage sites and molecular-sieving capabilities. (b) The electronic-state modulation is achieved through in situ N/S codoping, as directly evidenced by XPS, which is expected to enhance charge delocalization and create favorable adsorption sites. The convergence of the 2 aspects within the N-S@HDM-1300 architecture creates a synergistic platform designed to enhance Na^+^ storage (Fig. 3K), the electrochemical validation of which is provided in the following sections.

### Electrochemical properties of N-S@HDM

**Fig. 4.**
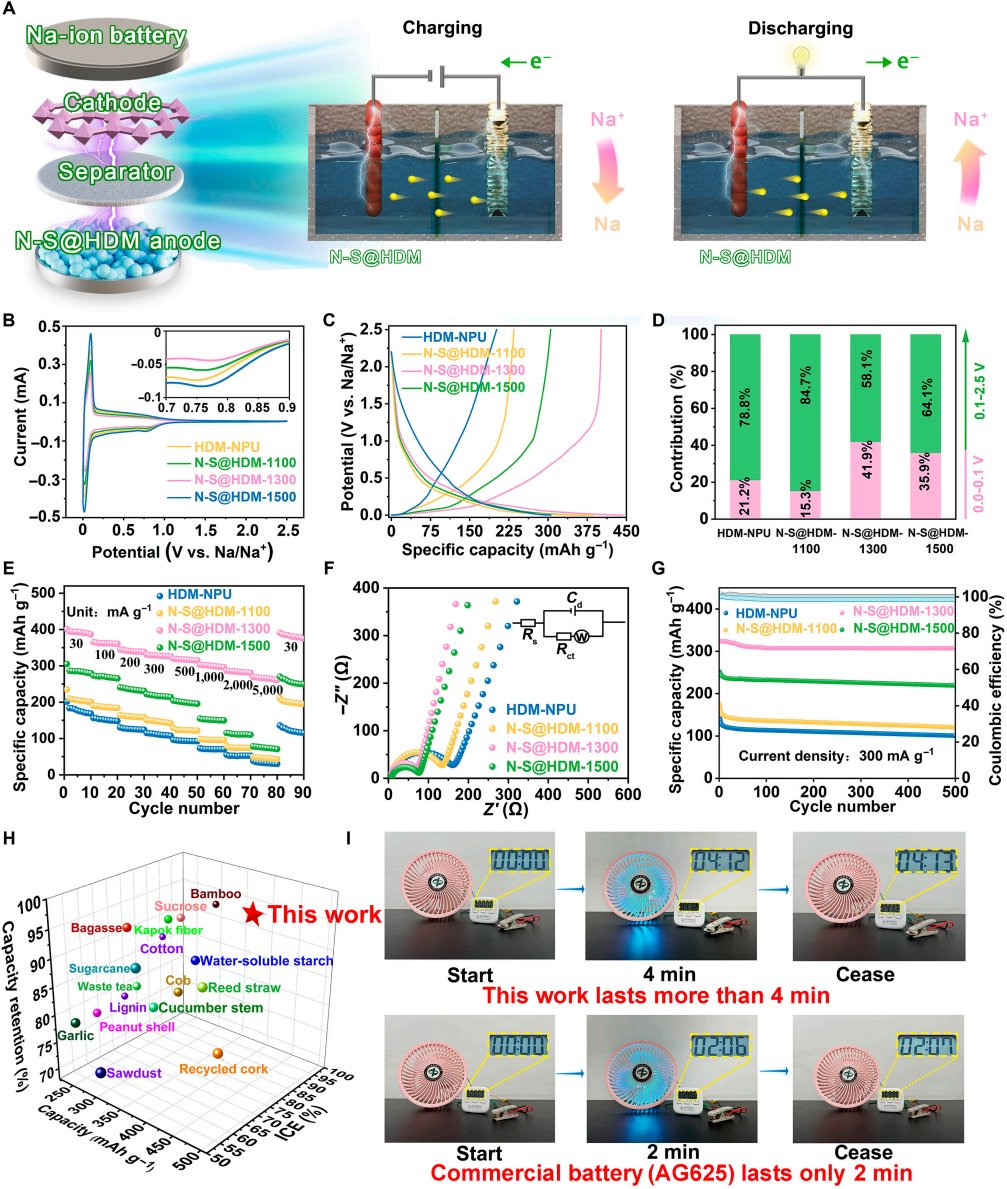
Electrochemical properties of the N-S@HDM-based half-cell. (A) Schematic of the sodium-ion battery (SIB) half-cell configuration, charge/discharge mechanisms, and potential applications. (B) Cyclic voltammetry (CV) curves at 0.1 mV s^−1^ (first cycle). (C) Initial galvanostatic charge/discharge (GCD) profiles at 30 mA g^−1^. (D) Capacity contribution analysis across different potential regions. (E) Rate performance. (F) Nyquist plots with the equivalent circuit (inset). (G) Cycling stability at 300 mA g^−1^. (H) Comparison of electrochemical properties between N-S@HDM-based SIBs and SIBs utilizing other biomass-derived carbons. (I) Operational endurance comparison between N-S@HDM-based SIBs and commercial AG625 batteries. ICE, initial coulombic efficiency.

N-S@HDM samples are anticipated to function as negative electrode materials for SIBs in a variety of application scenarios, including potential use in the energy-supplying devices of future bionic robots. Their electrochemical properties were assessed in a half-cell configuration, with sodium metal serving as the counter electrode, as illustrated in Fig. 4A. Cyclic voltammetry (CV) tests at 0.1 mV s^−1^ reveal sharp and symmetric oxidation/reduction peaks in all samples (Fig. 4B), corresponding to the reversible deintercalation/intercalation of sodium ions within the graphene layers. The irreversible broad peak near 0.75 V originates from electrolyte decomposition and SEI formation on the surface of carbon [39], with N-S@HDM-1300 exhibiting the weakest peak intensity in this region, verifying that the “synergistic ultramicropore-confined and electronic-state modulation strategy” effectively suppresses SEI growth. The galvanostatic charge/discharge (GCD) profiles (30 mA g^−1^) (Fig. 4C) indicate that the capacities of N-S@HDM-1100, N-S@HDM-1300, and N-S@HDM-1500 increase to 234.8, 401.5 and 305.1 mAh g^−1^, respectively, as compared to that of HDM-NPU (201.5 mAh g^−1^) obtained without preoxidation or urea mixing. Also, the capacity of N-S@HDM-1300 is higher than those of its singly modified counterparts, HDM-NU (without urea, 218.6 mAh g^−1^) and HDM-NP (without preoxidation, 316.4 mAh g^−1^) (Fig. S14 and Table S8). This result highlights the synergistic effect of combining preoxidation and urea treatment. In addition, at a mass loading of 2.0 mg cm^−2^, the N-S@HDM-1300 electrode delivers an areal capacity of 0.803 mAh cm^−2^. The superior property of N-S@HDM-1300 is due to the heteroatom doping-induced electrochemical active sites and the sodium storage space provided by its ultramicroporous structure [40].

The plateau capacity contribution is standardly defined and calculated based on the capacity in the voltage region below 0.1 V from the initial GCD profile. In comparison to HDM-NPU (ICE = 66.1%; plateau capacity contribution = 21.2%) and N-S@HDM-1100 (ICE =60.7%; plateau capacity contribution = 15.3%) as well as N-S@HDM-1500 (ICE =82.4%; plateau capacity contribution = 35.9%), N-S@HDM-1300 has optimized ICE (90.6%) and plateau capacity contribution (41.9%) (Fig. 4D). The ICE of N-S@HDM-1300 is also higher than those of HDM-NU (87.2%) and HDM-NP (58.2%) (Table S8). A comparison between HDM-NP (with urea mixing but no preoxidation) and HDM-NU (with preoxidation but no urea mixing) reveals that the absence of preoxidation contributes to a lower ICE. This can be attributed to the reduction in closed pore formation, which is known to adversely affect ICE. Meanwhile, N doping derived from urea markedly enhances the capacity, which is likely due to the introduction of additional active sites (as proved by XPS, Fig. S15 and Table S9) and the improved electrical conductivity. The sodium storage mechanisms in hard carbon mainly include (a) adsorption at pores/edges/heteroatoms, (b) intercalation between graphene layers, and (c) Na cluster formation within closed pores [7]. To conclusively verify the Na cluster formation in N-S@HDM-1300, we conducted ex situ characterizations on electrodes at the fully sodiated state (discharged to 0.01 V). XRD patterns (Fig. S16) indicate a distinct shift of the (002) peak to lower angles after sodiation (10 cycles), providing direct evidence of interlayer expansion due to Na^+^ intercalation. More importantly, SEM–EDS analysis (Fig. S17) demonstrates a remarkably high and uniform sodium content of 19.35 wt.% within the carbon matrix of the sodiated electrode, as compared to that of the pristine electrode (only 3.26 wt.%). The integration of interlayer expansion and such a high sodium content provides compelling evidence that sodium ions not only intercalate into the graphitic domains but also fill the ultramicropores, forming quasi-metallic sodium clusters, which is the origin of the substantial plateau capacity. The high ICE of N-S@HDM-1300 originates from its ultralow supermicropore/mesopore specific surface area (12.35 m^2^ g^−1^), reducing irreversible sodium consumption during SEI formation, while the exceptional plateau capacity arises from the ultramicropore-dominant Na^+^-filling mechanism [41].

When the current density gradually increases from 30 to 5,000 mA g^−1^, all electrodes exhibit capacity decay trends. Among them, N-S@HDM-1300 delivers a charge specific capacity of 265.0 mAh g^−1^ at 5,000 mA g^−1^ (Fig. 4E), with a capacity retention rate of 68.7%, markedly outperforming HDM-NPU (19.8%), N-S@HDM-1100 (21.9%), and N-S@HDM-1500 (25.6%). This superior rate capability stems from the optimized pore connectivity of N-S@HDM-1300, facilitating rapid Na^+^ diffusion kinetics within the hard carbon matrix, thus maintaining stable ion transport pathways under high current densities [42]. Nyquist plots further confirm this mechanism (Fig. 4F). The semicircular arcs observed in the mid-to-high frequency region arise from the combined effects of solution resistance (*R*_s_) and charge-transfer resistance (*R*_ct_), whereas the linear portion at low frequencies is associated with Warburg diffusion impedance (*Z*_w_). The Nyquist plots were fitted by using Randles equivalent circuits. In addition to *R*_s_, *R*_ct_, and *Z*_w_, a constant phase element was introduced to account for the nonideal capacitive behavior, as ideal capacitive responses are not always evident in experiments. The corresponding fitting parameters are summarized in Table S10. N-S@HDM-1300 shows the smallest semicircle diameter (*R*_ct_) and the steepest low-frequency slope (*Z*_w_), which demonstrate enhanced conductivity and Na^+^ diffusion rate.

During 500-cycle GCD tests at 300 mA g^−1^, N-S@HDM-1300 retains a discharge capacity of 307.3 mAh g^−1^ (95.0% capacity retention), remarkedly higher than those of HDM-NPU (70.9%), N-S@HDM-1100 (72.0%), and N-S@HDM-1500 (87.3%) (Fig. 4G). Its coulombic efficiency remains stable at 99.6% to 100%, demonstrating its exceptional reaction reversibility. This excellent cycling stability is attributed to the maximized N-G (graphitic nitrogen) content (2.59% by XPS quantification) under 1,300 °C pyrolysis, where the planar tricoordinated structure anchors nitrogen atoms to the carbon layers via strong covalent bonds, effectively suppressing the structural collapse during cycling [43]. Moreover, the abundant ultramicropores in N-S@HDM-1300 spatially confine the Na clusters, mitigating stress cracks induced by volume expansion. The electrochemical properties of HDM-NPU, as well as N-S@HDM-1100, N-S@HDM-1300, and N-S@HDM-1500, are summarized in Table S11. A performance comparison with other biomass-derived hard carbon (Fig. 4H and Table S12) reveals that N-S@HDM-1300 leads in key metrics: ICE (90.6%), specific capacity (401.5 mAh g^−1^), and cycling stability (95.0%). In addition, the specific capacity and ICE of N-S@HDM-1300 are superior to those of several representative commercial hard carbon materials (e.g., T-Na-HC01, MSE PRO, BSHC-300 BTR, and XW-C; Table S13). In actual application tests, the N-S@HDM-1300-based SIB powers a 15 W fan for 4 min and 13 s, nearly double the runtime of the commercial AG625 battery (2 min and 7 s) (Fig. 4I and Movies S1 and S2, Supplementary Materials), proving its commercial potential.

**Fig. 5.**
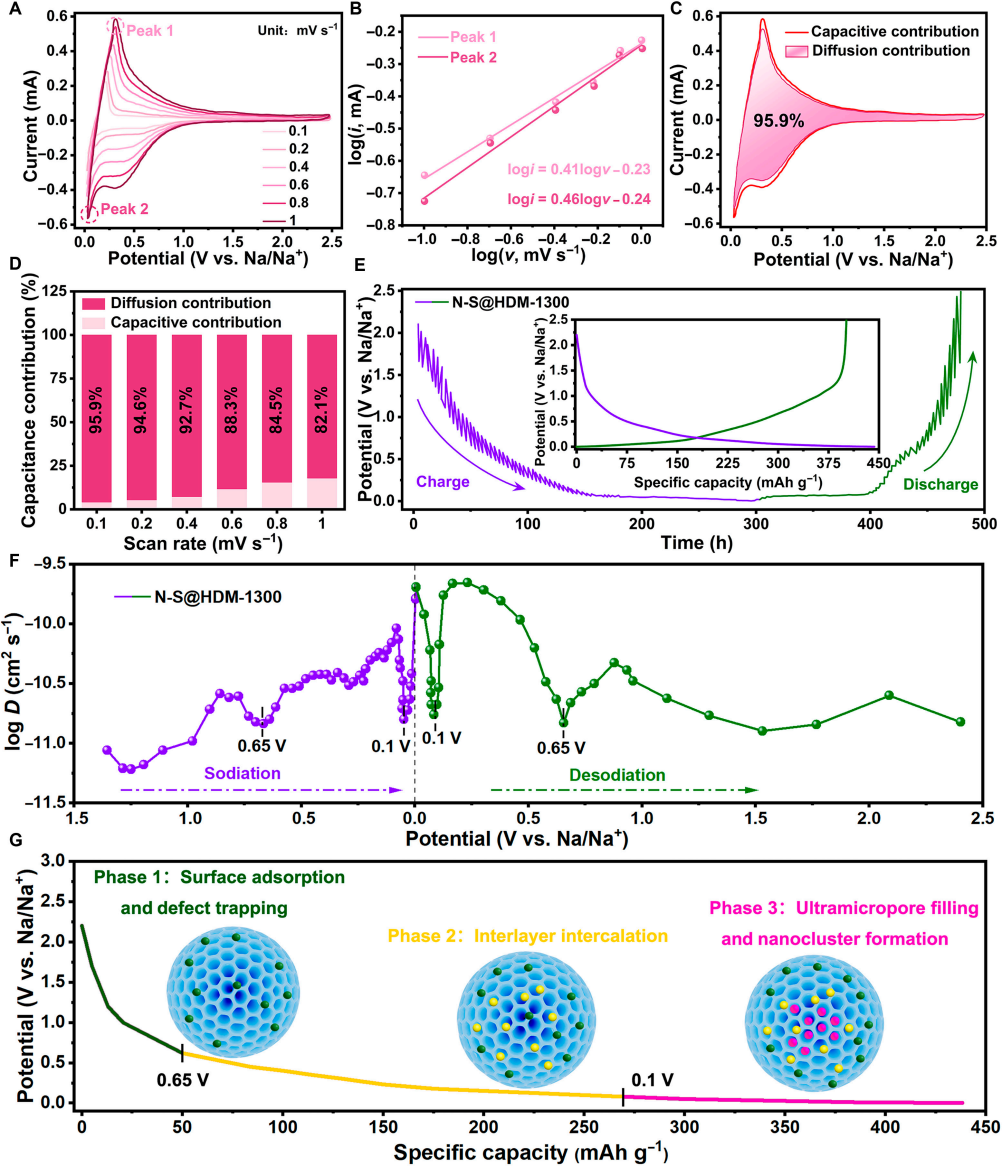
Reaction kinetics and sodium storage mechanisms of the N-S@HDM-1300-based SIBs. (A) CV curves at various scan rates. (B) Logarithmic fitting plots of peak current (*i*) versus scan rate (*v*) for the oxidation peak (peak 1, 0.25 V) and reduction peak (peak 2, 0.003 V). (C) Fitting result of capacitive/diffusion contribution separation based on the CV curve at 0.1 mV s^−1^ (the purple region represents a diffusion contribution ratio of 95.9%). (D) Histogram of diffusion-controlled capacity proportions at different scan rates. (E) Galvanostatic intermittent titration technique (GITT) profile with the corresponding GCD profile (inset). (F) Logarithmic plot of *D*_Na_^+^ variation versus potential during sodiation/desodiation. (G) Charging curve and schematic illustration of the sodium storage mechanism.

The reaction kinetics of the N-S@HDM-1300-based SIBs were investigated through CV at scan rates ranging from 0.1 to 1 mV s^−1^ (Fig. 5A). Based on the theoretical framework of Dunn’s method [44], the sodium storage mechanism was analyzed using the power-law relationship between peak current (*i*) and scan rate (*v*):

$$i=a\times v^{b}$$ (1)

$$\text{log}\left(i\right)=b\text{log}\;\left(v\right)+\text{log}\;\left(a\right)$$ (2)

The linear regression of log(*i*) versus log(*v*) (Fig. 5B) yields *b* values of 0.41 and 0.46 for the oxidation peak (peak 1, ~0.25 V) and reduction peak (peak 2, ~0.003 V), respectively. The *b* values, being markedly closer to 0.5 than 1.0, indicate that the sodium storage behavior within the low-voltage plateau is predominantly governed by a diffusion-controlled intercalation/deintercalation process, rather than surface capacitive effects [45]. To quantitatively decouple the contributions of diffusion and capacitance, a segmented current deconvolution equation is employed [46]:

$$i\left(V\right)=k_{1}v+k_{2}v^{1/2}$$ (3)

Here, *k*_1_*v* represents the rapid capacitive response from electric double-layer capacitance and surface redox reactions, while *k*_2_*v*^1/2^ corresponds to the diffusion-controlled faradaic processes in the bulk phase. By reformulating the equation as *i*/*v*^1/2^ = *k*_1_*v*^1/2^ + *k*_2_, the parameters *k*_1_ and *k*_2_ are fitted at varying potentials. As shown in Fig. 5C, the diffusion-controlled contribution (purple-shaded area) accounts for 95.9% of the total current at 0.1 mV s^−1^, highlighting the dominance of bulk sodium storage. Even as the scan rate increases to 1 mV s^−1^ (Fig. 5D), the diffusion-driven proportion keeps above 82%, revealing that the N-S@HDM-1300-based SIBs retain the battery-type energy storage features (centered on reversible Na^+^ intercalation) under high-rate conditions. This unique kinetic behavior is intrinsically associated with its special pore architecture: rich ultramicropores provide bulk sodium storage sites (diffusion-limited), while the scarcity of supermicropores and mesopores minimizes surface adsorption capacity, thereby optimizing the trade-off between capacity and rate capability.

The sodium-ion diffusion coefficient (*D*_Na_^+^, cm^2^ s^−1^) of the N-S@HDM-1300-based SIBs was assessed using the galvanostatic intermittent titration technique (GITT), where a pulsed current of 0.1 C (applied for 0.5 h) alternated with a 3-h relaxation period. The high consistency between the GITT and GCD profiles (Fig. 5E) validates the reliability of the testing protocol. The *D*_Na_^+^ value is calculated using a simplified form of Fick’s second law [47]:

$$D_{{\mathrm{Na}}^{+}}=\frac{4}{\pi \tau }{\left(\frac{m_{B}V_{M}}{M_{B}S}\right)}^{2}{\left(\frac{\Delta E_{s}}{\Delta E_{\tau }}\right)}^{2}$$ (4)

Here, *τ* denotes the pulse duration (s), *m*_B_ is the mass of the active material (g), *V*_M_ is the molar volume of the active material (cm^3^ mol^−1^), *M*_B_ is its molecular weight (g mol^−1^), *S* is the specific surface area (m^2^ g^−1^), Δ*E*_s_ is the equilibrium open-circuit potential change induced by the current pulse, and Δ*E_τ_* is the total transient potential variation after *iR* compensation. Analysis of the log*D* vs. potential curve during sodiation/desodiation (Fig. 5F) reveals a pronounced inflection near 0.65 V, attributed to (a) the dynamic evolution of interfacial impedance during initial SEI formation and (b) the transition in sodium storage mechanisms from surface/defect adsorption (high-potential region, above 0.65 V) to graphite interlayer intercalation (mid-potential region, 0.1 to 0.65 V). Additionally, the abrupt change near 0.1 V corresponds to the pore filling of sodium clusters within ultramicropores (below 0.7 nm), where the confined pore geometry elevates diffusion barriers. Remarkably, the SIB device exhibits superior *D*_Na_^+^ values (above 10^−11^ cm^2^ s^−1^) across the entire potential range, as compared to other reports [48,49], benefiting from its unique structural merits: (a) the highly interconnected ultramicroporous network (94.27% pore volume fraction) shortens ion transport pathways, and (b) N/S codoping induces an expanded interlayer spacing (0.389 nm), reducing the intercalation energy barriers. These structural innovations synergistically enhance both ionic and electronic conductivities, positioning N-S@HDM-1300 as an ideal anode for high-rate SIBs.

### Full-cell performance

**Fig. 6.**
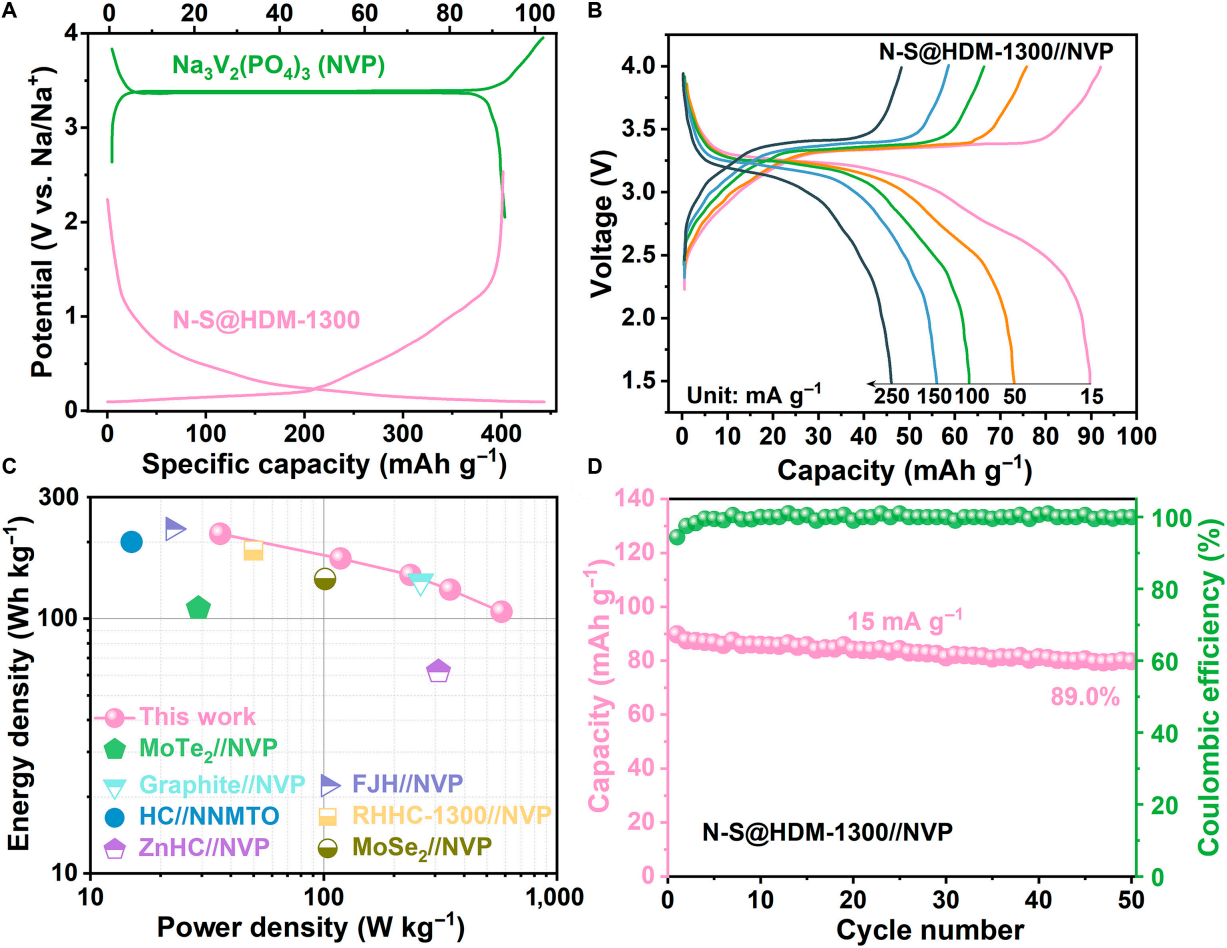
Electrochemical properties of the N-S@HDM-1300//NVP full cell. (A) GCD curve of the Na_3_V_2_(PO_4_)_3_ (NVP) cathode at 30 mA g^−1^. (B) GCD profiles of the full cell under various current densities. (C) Ragone plot of the full cell in comparison with recent biomass-derived hard carbon-based SIB full cells. (D) Cycling performance of the full cell at 15 mA g^−1^. MoTe_2_//NVP, 2-dimensional layered structure MoTe_2_//NVP; graphite//NVP, regulating ternary graphite intercalation compounds//NVP; HC//NNMTO, magnesium gluconate and glucose-based hard carbon//NNMTO; ZnHC//NVP, zinc-doped hard carbon//NVP; FJH//NVP, anthracite flash Joule heating preparation of hard carbon//NVP; RHHC-1300//NVP, rice-husk-derived hard carbon//NVP; MoSe_2_//NVP, MoSe_2_-covered N/P-doped carbon nanosheets//NVP.

To assess the practical application potential of the N-S@HDM-1300 anode in SIBs, we assemble a full cell by pairing it with a Na_3_V_2_(PO_4_)_3_ (NVP) cathode, denoted as the N-S@HDM-1300//NVP full cell. A glass fiber separator was used, and the electrolyte consisted of 1 M NaPF_6_ dissolved in a mixture of dimethyl ether glycolate (DEG) and dimethyl ether (DME). The NVP cathode shows a characteristic discharge plateau at approximately 3.4 V (vs. Na^+^/Na) (Fig. 6A). The negative-to-positive capacity ratio is controlled at approximately 1.1, which aims to maximize the energy density of the full cell while ensuring operational safety by providing a sufficient sodium reservoir.

As shown in Fig. 6B, the full cell displays a flat and stable charge/discharge plateau around 3.3 V. This observed voltage aligns well with the characteristic plateau of the NVP cathode, indicating stable operating conditions for the battery. The full cell exhibits a reversible capacity of 89.7 mAh g^−1^ at 15 mA g^−1^, calculated based on the cathode mass as it acts as the sole sodium source. With an average discharge voltage of ~3.0 V, the device achieves an energy density of 215.5 Wh kg^−1^ at a power density of 36.0 W kg^−1^ (based on total active mass, cathode:anode mass ratio ≈ 4:1). As shown in Fig. 6C and Table S14, this performance is highly competitive compared with those of some recently reported SIBs. Furthermore, the full cell demonstrates an excellent rate capability (Fig. 6B), retaining a capacity of 46.0 mAh g^−1^ (51.3% retention) even at a high current density of 250 mA g^−1^. This result directly corroborates the rapid sodium-ion diffusion kinetics of N-S@HDM-1300, as revealed in the half-cell tests. The origin of this superior kinetics can be traced to the well-connected ion transport pathways enabled by the high amphiphilic factor (*f*_a_ = 0.85) and the reduced charge-transfer resistance resulting from heteroatom doping.

More importantly, the full cell exhibits superior cycling stability. It maintains a capacity retention of 89.0% after 50 cycles (Fig. 6D). We attribute this cyclability to the closed-pore-dominated structure (94.27%) of N-S@HDM-1300. This specific pore architecture effectively suppresses continuous electrolyte decomposition and the growth of a thick SEI during cycling (Fig. S18). The advantage responsible for the high ICE in half-cells is effectively carried over to the full-cell configuration, leading to a high coulombic efficiency and outstanding capacity retention.

## Discussion

**Fig. 7.**
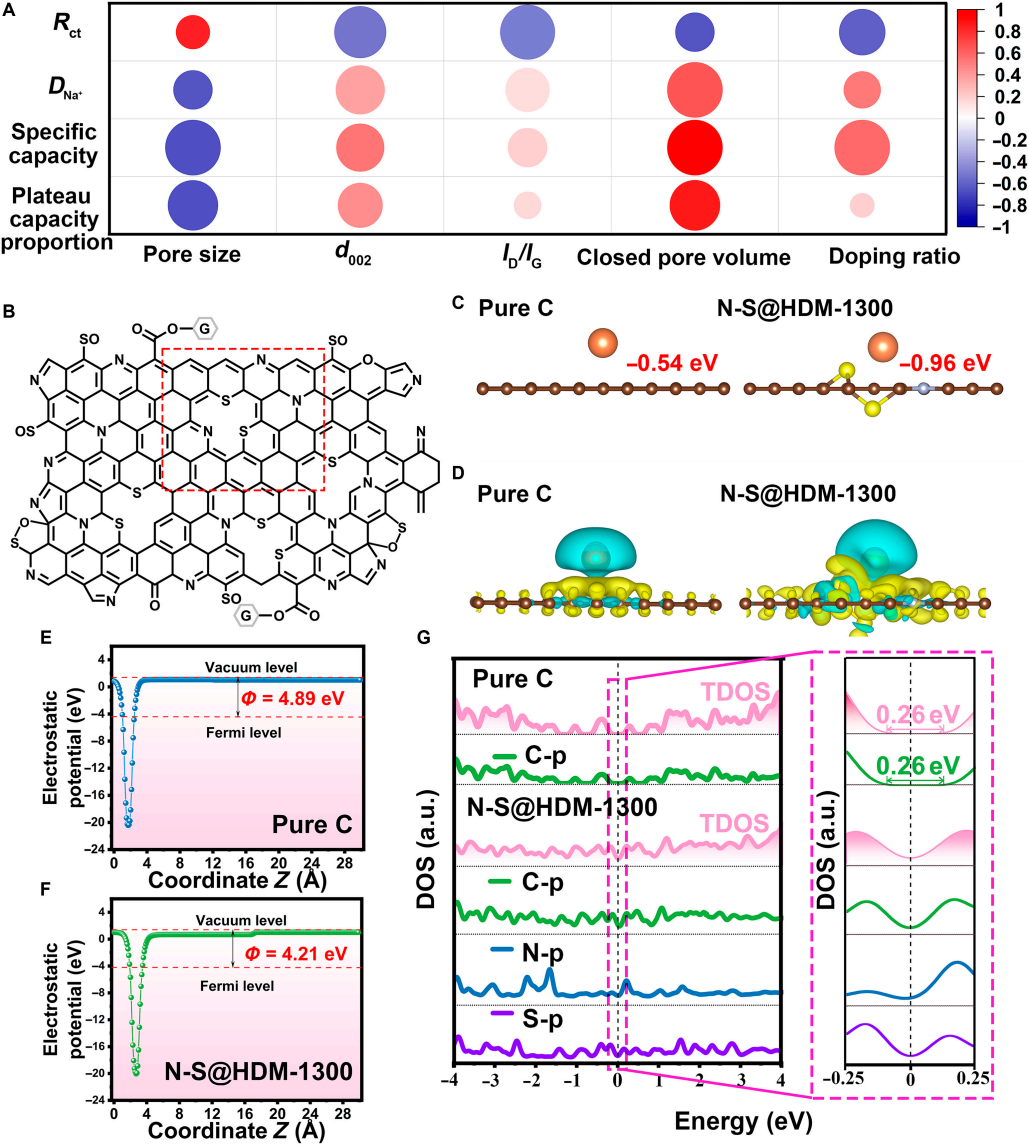
Unraveling the synergistic effects of ultramicropore confinement and electronic-state modulation on sodium storage in N-S@HDM-1300. (A) Heatmap illustrating the correlation between the physical properties (pore size, *d*_002_, *I*_D_/*I*_G_ ratio, closed pore volume, and doping ratio) and electrochemical properties (*R*_ct_, plateau capacity proportion, *D*_Na_^+^, and specific capacity) of N-S@HDM-1300. (B to G) density functional theory (DFT) analysis: (B) proposed atomic model of N-S@HDM-1300, constructed from experimental EDS/XPS data (the red box highlights the representative unit used for Na^+^ interaction studies); (C) optimized geometries of Na^+^ adsorbed on pure C and N-S@HDM-1300, with their respective adsorption energies (*E*_ads_); (D) 3-dimensional charge density difference at the interface between Na^+^ and N-S@HDM-1300/pure C; (E and F) planar-averaged electrostatic potential profiles along the surface normal (*Z*-axis); (G) comparison of the densities of states (DOSs) of pure C and N-S@HDM-1300. TDOS, total DOS.

To unravel the synergistic effects of ultramicropore confinement and electronic-state modulation on sodium storage in N-S@HDM-1300, a detailed correlation analysis between physical properties (pore size, *d*_002_, *I*_D_/*I*_G_ ratio, closed pore volume, and doping ratio) and electrochemical properties (*R*_ct_, plateau capacity proportion, *D*_Na_^+^, and specific capacity) is elucidated through heatmaps (Fig. 7A and Fig. S19a_1_ to e_4_). The heatmaps clearly indicate that *R*_ct_ is negatively (blue) correlated with *d*_002_, *I*_D_/*I*_G_ ratio, closed pore volume, and doping ratio. Conversely, *D*_Na_^+^, specific capacity, and plateau capacity proportion are positively (red) correlated with the 4 parameters. A larger diameter and a darker color of circles indicate a higher effect size (steeper slope) and a stronger correlation (higher *R*^2^ value). Clearly, the pore feature (pore size and closed pore volume) plays an important role in *D*_Na_^+^, specific capacity, and plateau capacity proportion. These results indicate that the reduced pore size and increased closed pore volume in HDM materials can substantially improve sodium-ion storage and diffusion kinetics, highlighting the key role of ultramicropore confinement. The *d*_002_ value has a marked impact on both *R*_ct_ and *D*_Na_^+^ since it reflects the degree of graphitization and the space available for the intercalation/deintercalation of Na^+^ ions. The *I*_D_/*I*_G_ ratio is a crucial indicator of the disorder degree in carbon materials and is hence directly related to *R*_ct_. Regarding the doping ratio, not only is the specific capacity markedly affected by the increased adsorption sites resulting from N–S codoping, but *R*_ct_ is also tightly related to the doping ratio. This validates that N–S codoping effectively modulates the electronic state of the material.

The electronic-state modulation induced by N–S codoping was further investigated using DFT. Heteroatom doping, a vital strategy for tailoring carbon skeleton, involves substituting specific carbon lattice atoms with heteroatoms (like N and S). This substitution causes a redistribution of charge and spin density within the carbon matrix due to differences in electronegativity. This effectively modulates the carbon’s work function, enhances Na^+^ adsorption at targeted sites, and establishes efficient active sites for improved Na^+^ interaction [50]. The computational model is rigorously constructed to align with experimental data (EDS and XPS), incorporating precise elemental compositions (C, O, S, and N) and chemical bonding configurations (e.g., C–O, C=O, COOR, C–S, O–S, N–O, N–5, and N–6) (Tables S15 and S16). The Open Babel software was employed to randomize the distribution of functional groups in the DFT model, with a representative unit (red box, Fig. 7B) selected to investigate the interaction between N-S@HDM and Na^+^. Initially, we built various doping models to identify the optimized geometric configurations (Fig. S20). Among these, type c is proposed to be the most energetically stable due to its lowest formation energy (−8.77 eV). Consequently, the type c configuration is selected to study the impact of N–S codoping. The adsorption energy (*E*_ads_) of Na^+^ on the surfaces of electrodes is expressed as [51]

$$E_{\mathrm{ads}}=E_{\mathrm{Na}/\text{surf}}-E_{\text{surf}}-E_{\mathrm{Na}\left(g\right)}$$ (5)

where *E*_Na/surf_ is the energy of Na^+^ adsorbed on the surfaces of electrodes, *E*_surf_ is the energy of the clean electrode surface, and *E*_Na(g)_ is the energy of the isolated Na^+^ in a cubic periodic box with a 20-Å side length. *E*_ads_ reveals a stronger binding affinity for Na^+^ on N-S@HDM-1300 (−0.96 eV) compared with pure C (−0.54 eV) (Fig. 7C). Electron density difference maps (Fig. 7D) further illustrate the expanded electron-rich (yellow) and electron-deficient (blue) regions in the N-S@HDM-1300/Na^+^ system, corroborating accelerated charge-transfer kinetics [52].

The work function (*Φ*), the minimum energy needed to liberate an electron from a solid’s surface, indicates the energy surface electrons require to transfer across the electrode–electrolyte interface. The work function (*Φ*) is expressed as

$$\Phi =E_{\mathrm{vac}}-E_{f}$$ (6)

where *E*_vac_ and *E*_f_ are the electrostatic potentials of the vacuum level and Fermi level, respectively. Work function analysis highlights a reduced *Φ* value for N-S@HDM-1300 (4.21 eV vs. 4.89 eV for pure C), ascribed to heteroatom-induced electron delocalization, which facilitates spontaneous electron migration and Fermi level equilibration (Fig. 7E and F). Density of states profiles (Fig. 7G) demonstrate a semiconductor-to-conductor transition in N-S@HDM-1300, evidenced by a nonzero density of states at the Fermi level, whereas pure C exhibits the characteristics of a semiconducting behavior with a bandgap of 0.26 eV. This result underscores the critical role of heteroatoms in modulating the electronic state of the carbon skeleton and establishing efficient electron transport pathways for rapid Na^+^ intercalation/deintercalation.

## Conclusion

This study demonstrates a breakthrough in SIB anode materials by synergistically integrating the ultramicropore confinement and electronic-state modulation strategies in sustainable lignin-derived hard carbon. Utilizing SLS, a paper-making byproduct, as a precursor, N/S-codoped hard carbon microspheres (N-S@HDM) are synthesized through preoxidation-induced covalent cross-linking and optimized pyrolysis. The 300 °C air preoxidation step is critical for forming a 3D molecular network, suppressing graphitic microcrystal alignment, and expanding interlayer spacing while simultaneously generating closed ultramicropores (94.27% closed pore ratio at 1,300 °C). This pore architecture enables molecular sieving to block solvent co-intercalation, minimizing irreversible electrolyte decomposition and achieving an exceptional ICE of 90.6%. Concurrently, S self-doping and urea-derived N doping optimize the electronic structure by enhancing electron delocalization, reducing charge-transfer resistance, and creating high-density Na^+^ adsorption sites. The synergistic effects of these strategies endow N-S@HDM-1300 with a superb reversible capacity of 401.5 mAh g^−1^ at 0.03 A g^−1^ and outstanding cyclability (95.0% capacity retention after 500 cycles), surpassing those of most biomass-derived hard carbons. This work not only resolves the long-standing trade-off between ICE and capacity in SIB anodes but also highlights the potential of sustainable lignin valorization for high-performance energy storage systems, offering a scalable and eco-friendly pathway for next-generation battery technologies.

## Materials and Methods

### Materials

SLS was purchased from Sinopharm Chemical Reagent Co., Ltd. (China). Urea, sodium hexafluorophosphate solution (NaPF_6_), conductive carbon black (Super P), hydrochloric acid (HCl, analytical reagent grade), ethanol, carboxymethylcellulose, DEG, DME, and NVP were provided by Macklin Biochemical Technology Co., Ltd.

### Preparation of N-S@HDM

SLS was put into a muffle furnace for preoxidation treatment under an air atmosphere: heated from 20 to 300 °C at 3 °C min^−1^, kept at 300 °C for 2 h, and naturally cooled to gain the preoxidized intermediate (POSLS). The resultant POSLS was then subjected to successive washing with 1 wt.% HCl, ethanol, and distilled water to remove sodium ions and other impurities, followed by drying at 80 °C. The dried POSLS was then uniformly mixed with urea at a mass ratio of 2:1 in a porcelain boat, then placed in a nitrogen-protected tube furnace, heated to target temperatures (1,000, 1,300, and 1,500 °C) at 2 °C min^−1^, and kept at each temperature for 2 h before natural cooling. The carbonized products were sequentially washed, vacuum-dried, and labeled as N-S@HDM-*X* (where *X* denotes the target temperature). To evaluate the preoxidation effect, a control group was prepared without preoxidation; that is, the SLS precursor without mixing with urea was directly carbonized at 1,300 °C. The resultant product was named HDM-NPU. Furthermore, 2 additional control samples were prepared and designated as HDM-NP and HDM-NU, respectively. HDM-NP was prepared by directly pyrolyzing the SLS precursor mixed with urea at 1,300 °C without preoxidation. In contrast, HDM-NU was obtained by pyrolyzing POSLS at 1,300 °C in the absence of urea. The key details on the raw material, intermediates, and resulting carbon materials are summarized in Table1.

**Table 1.** Summary of sample abbreviations and corresponding preparation conditions

Sample	Preoxidation	Pyrolysis with urea at 1,300 °C	Pyrolysis without urea at 1,300 °C
SLS (feedstock)			
Preoxidized SLS (POSLS)	√		
POSLS-derived HDM prepared with urea (N-S@HDM)	√	√	
SLS-derived HDM prepared without preoxidation and urea (HDM-NPU)			√
POSLS-derived HDM prepared without urea (HDM-NU)	√		√
SLS-derived HDM prepared without preoxidation but with urea (HDM-NP)		√	

HDM, hard carbon microsphere

### Characterizations

Raman analysis was performed using a Raman spectrometer (HORIBA Scientific LabRAM HR Evolution, Japan). XPS was performed on a Thermo Fisher Nexsa spectrometer (USA). XRD patterns were collected on an x-ray diffractometer (Bruker-D8 Advance, Germany). The microstructures, elemental compositions, and SAED patterns were characterized by SEM (Hitachi Regulus 8230, Japan), TEM (JEOL JEM-2100 Plus, Japan), and EDS. Surface area and porosity were determined via nitrogen and carbon dioxide adsorption analyses (MicrotracBEL, BELSORP Max II, Japan) at 77 and 273 K, respectively. The true density of samples was measured by using a helium pycnometer (MicrotracBEL, BELPycno, Japan). FTIR spectra were recorded on a Shimadzu IRTracer-100 spectrometer (Japan). Electrical conductivity was tested using a 4-probe resistivity system (RTS-9, Guangzhou Four-Probe Technology Co., Ltd.). The analysis of pores in hard carbon was conducted by using SAXS on an Ultima IV x-ray diffractometer (Rigaku). *f*_a_, which quantifies the interplay between short-range order and long-range disorder in porous networks, was derived from the SAXS data. The calculation method for *f*_a_ is detailed in the Supplementary Materials. The high-resolution solid-state ^13^C NMR spectra were recorded on Bruker AVANCE-600 using a 5-mm TXI cryoprobe. TG analysis coupled with MS (Thermo plus EVO2, Rigaku) was applied to study the evolution of the SLS structure during preoxidation.

### Electrochemical tests

By mixing the as-prepared carbon materials, Super P, and carboxymethylcellulose binder at a mass ratio of 8:1:1 and subsequently grinding the mixture, the electrode slurry was prepared. The slurry was uniformly coated on a 5-μm-thick copper foil current collector, vacuum-dried at 80 °C for 12 h to fabricate the electrode sheet. The electrode diameter was 8 mm, which corresponds to an area of ~0.5 cm^2^. The mass loading of the active material was controlled at ~2.0 mg cm^−2^. A CR2032 coin cell was constructed in an inert atmosphere glove box (H_2_O/O_2_ < 0.1 ppm), using sodium foil as the counter electrode, a Whatman GF/F glass fiber separator, and an electrolyte of 1 M NaPF_6_ in equimolar DEG/DME. Electrochemical tests included GCD on a LAND-CT 3001A battery tester, as well as electrochemical impedance spectroscopy and CV through a Wuhan CorrTest CS3104 system. For the assembly of full cells, we paired the carbon anode with an NVP cathode, coded as the N-S@HDM-1300//NVP full cell. A glass fiber separator was used, and the electrolyte consisted of 1 M NaPF_6_ dissolved in a mixture of DEG/DME. The electrochemical testing procedures for the full cells were similar to those used for the half cells.

## Data Availability

The data that support the findings of this study are available within this paper and/or included in the Supplementary Materials and from the corresponding author upon request. Source data are provided with this paper.
