# Supplementary material for: Synergistic Ultramicropore-Confined and Electronic-State Modulation Strategies in Sustainable Lignin-Derived Hard Carbon for Robust Sodium-Ion Batteries
Source: Research (Wash D C). 2026 Jan 15;9:1039. doi: 10.34133/research.1039 (PMC12804597; doi:10.34133/research.1039)
Supplement: Supplementary 1 — Figs. S1 to S20 Tables S1 to S16 Movies S1 and S2 [file research.1039.f1.zip › Supporting information for Research.docx]

**Supporting Information**

**Synergistic ultramicropore-confined and electronic-state modulation strategies in sustainable lignin-derived hard carbon for robust sodium-ion batteries**

Yuzhong Xie,^a^ Yuqing Wang,^a^ Yusuke Yamauchi,^b,c^ Minjun Kim,^b,c^ Fang Yuan,^a^ Yuhang He,^a^ Yiqiang Wu,^a^ Caichao Wan^a^*

*^a^College of Materials and Energy, Central South University of Forestry and Technology, Changsha 410004,* ***P. R.*** *China.*

*^b^Australian Institute for Bioengineering and Nanotechnology (AIBN), The University of Queensland, Brisbane, QLD 4072, Australia.*
*^c^Department of Materials Process Engineering, Graduate School of Engineering, Nagoya University, Nagoya 464-8603, Japan.*

**Corresponding author.*

*E-mail:* [*wancaichaojy@163.com*](mailto:wancaichaojy@163.com) *(C. W.)*

**Table of contents**

1. Comparative analysis of deconvoluted peak proportions in XPS O 1s spectra of SLS before and after the preoxidation (Table S1)4

2. XRD patterns of preoxidized and unpreoxidized SLS-derived carbon (Figure S1)5

3. TEM microstructural evolution from the unpreoxidized to preoxidized carbon (Figure S2)6

4. Raman spectra of preoxidized and unpreoxidized SLS-derived carbon (Figure S3) 7

5. N_2_/CO_2_ adsorption‒desorption isotherms and pore characteristic parameters of preoxidized and unpreoxidized SLS-derived carbon (Figures S4-S5 & Table S2)8

6. XPS survey spectra of N-S@HDM-1100, -1300, and -1500 (Figure S6)10

7. Comparative analysis of deconvoluted peak proportions in the XPS C 1s, N 1s, and S 2p spectra of N-S@HDM-1100, -1300, and -1500 (Table S3)11

8. Four-probe tests of electrode conductivity in N-S@HDM-1100, -1300, and -1500 (Figure S7)13

9. Four-probe tests of electrode conductivity in N-S@HDM-1300 and HDM-NPU (Figure S8)14

10. Tensile stress‒strain curves of N-S@HDM-1100, -1300, and -1500 (Figure S9)15

11. Elemental contents of N-S@HDM-1100, -1300, and -1500 (Table S4)16

12. Calculation of crystalline size (*D*) and interplanar spacing (*d*_002_) (Figure S10 & Table S5)17

13. Formation mechanism of closed ultramicropores (Figure S11)19

14. Raman spectra of N-S@HDM-1100, -1300, and -1500 (Figure S12)20

15. N_2_ adsorption‒desorption isotherms of N-S@HDM-1100, -1300, and -1500 (Figure S13)21

16. Pore characteristic parameters of N-S@HDM-1100, -1300, and -1500 (Table S6)22

17. An approach for fitting fa in SAXS patterns utilizing the Teubner-Strey model 23

18. Ture density and porosity of N-S@HDM-1100, -1300, and -1500 (Table S7)24

19. Electrochemical properties of N-S@HDM-1300, HDM-NPU, HDM-NU, and HDM-NP (Figures S14 & Table S8)25

20. XPS survey spectra of SLS, POSLS, N-S@HDM-1300, HDM-NPU, HDM-NU, and HDM-NP (Figures S15 & Table S9)26

21. Evidences for the existence of sodium clusters (Figures S16-S17)27

22. Fitted parameters of the Randles equivalent circuit for Nyquist plots (Table S10)29

23. A summary of electrochemical properties for HDM-NPU, N-S@HDM-1100, -1300, and -1500 (Table S11)30

24. Electrochemical performance comparison of SIBs using 18 biochar variants (Table S12)31

25. Electrochemical properties of commercial hard carbon (Table S13)33

**26. Operational endurance demonstration: comparative analysis with commercial AG625 batteries (Movies S1-S2)34**

**27. Comparison of energy and power density of the assembled sodium-ion full-cell (Table S14)35**

**28. HRTEM images of the SEI film (Figure S19)36**

**29. Correlation between physical properties and electrochemical properties for N-S@HDM-1300 (Figure S18)37**

**30. Contents of C, O, S, and N elements and their bonds in the DFT model (Tables S15-S16)38**

**31. Computational details on the density functional theory (DFT) analysis (Figure S20)40**

**Supporting references42**

**1. Comparative analysis of deconvoluted peak proportions in XPS O 1s spectra of SLS before and after the preoxidation**

**Table S1.** Comparative analysis of deconvoluted peak proportions in the XPS O 1s spectra of SLS before and after the preoxidation.

| **O 1s** | | | | | |
| --- | --- | --- | --- | --- | --- |
| **Samples** | O‒H | C‒O | C=O | C(O)O |  |
| **SLS** | 8.23% | 18.23% | 58.08% | 15.46% |  |
| **POSLS** | 12.08% | 6.87% | 62.43% | 18.62% |  |

**2. XRD patterns of preoxidized and unpreoxidized SLS-derived carbon**


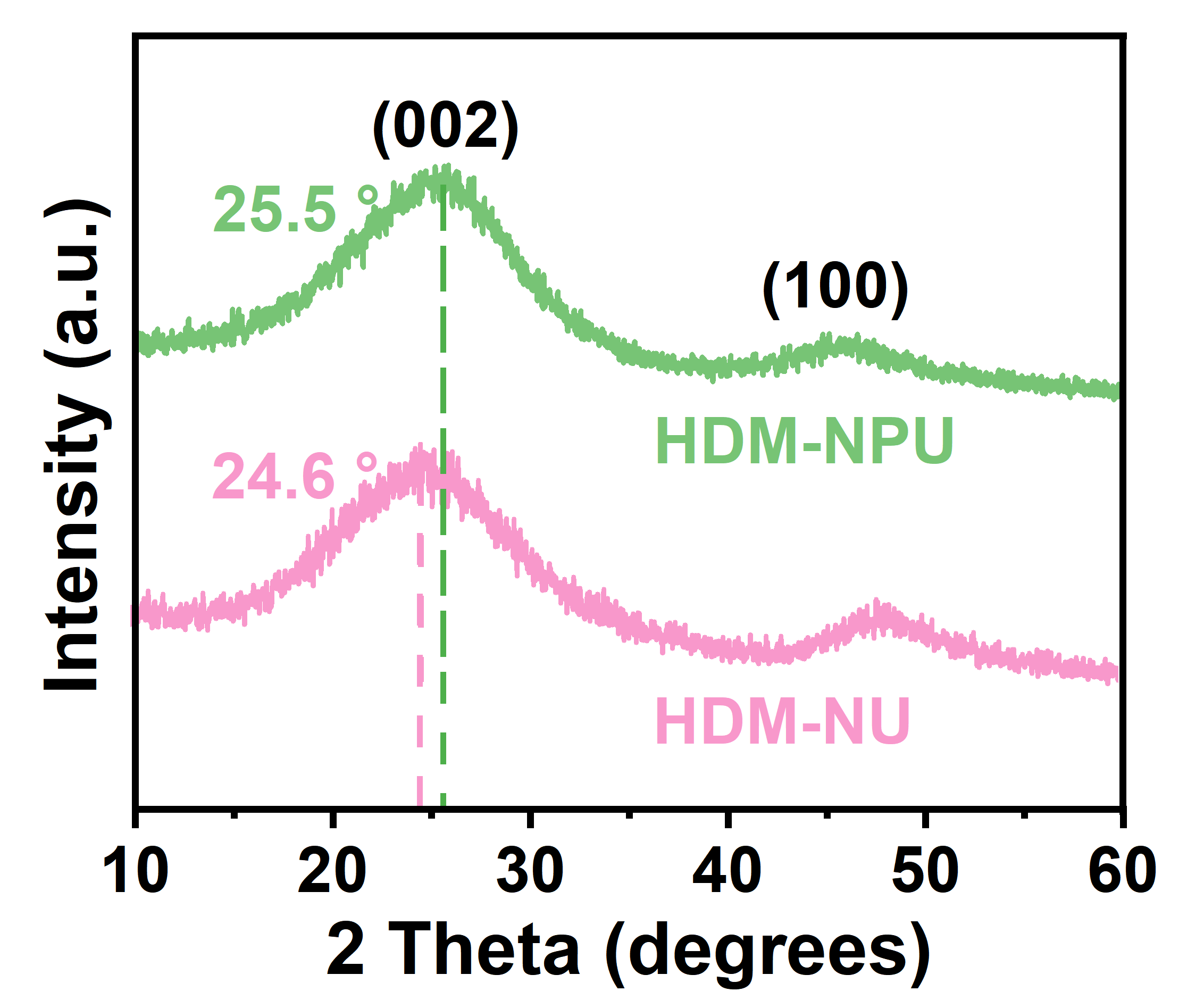


**Figure S1.** XRD patterns of the unpreoxidized and preoxidized carbon (HDM-NPU and HDM-NU).

**3. TEM microstructural evolution from the unpreoxidized to preoxidized carbon**


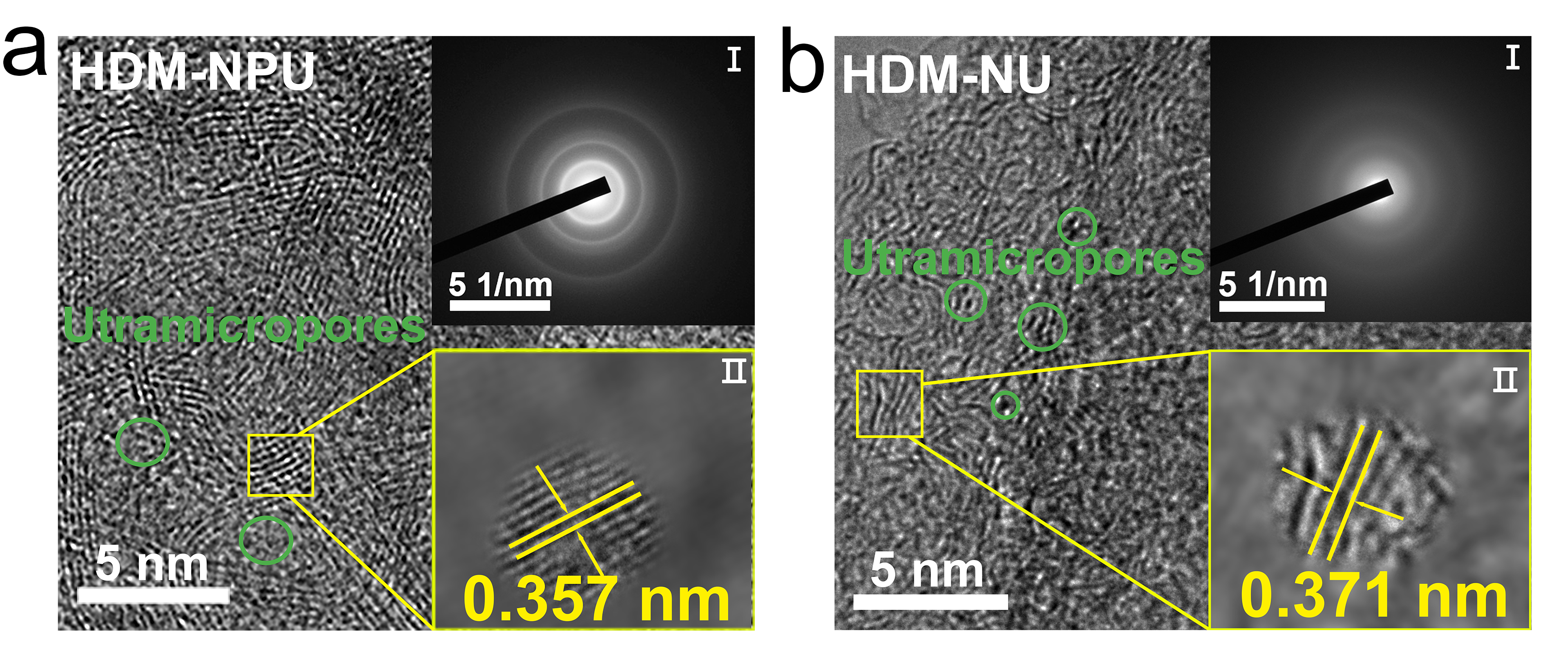


**Figure S2**. TEM microstructural evolution from the (a) unpreoxidized to (b) preoxidized carbon (HDM-NPU to HDM-NU). Insets: lattice-resolved HRTEM images and corresponding SAED patterns confirming interlayer expansion.

**4. Raman spectra of preoxidized and unpreoxidized SLS-derived carbon**


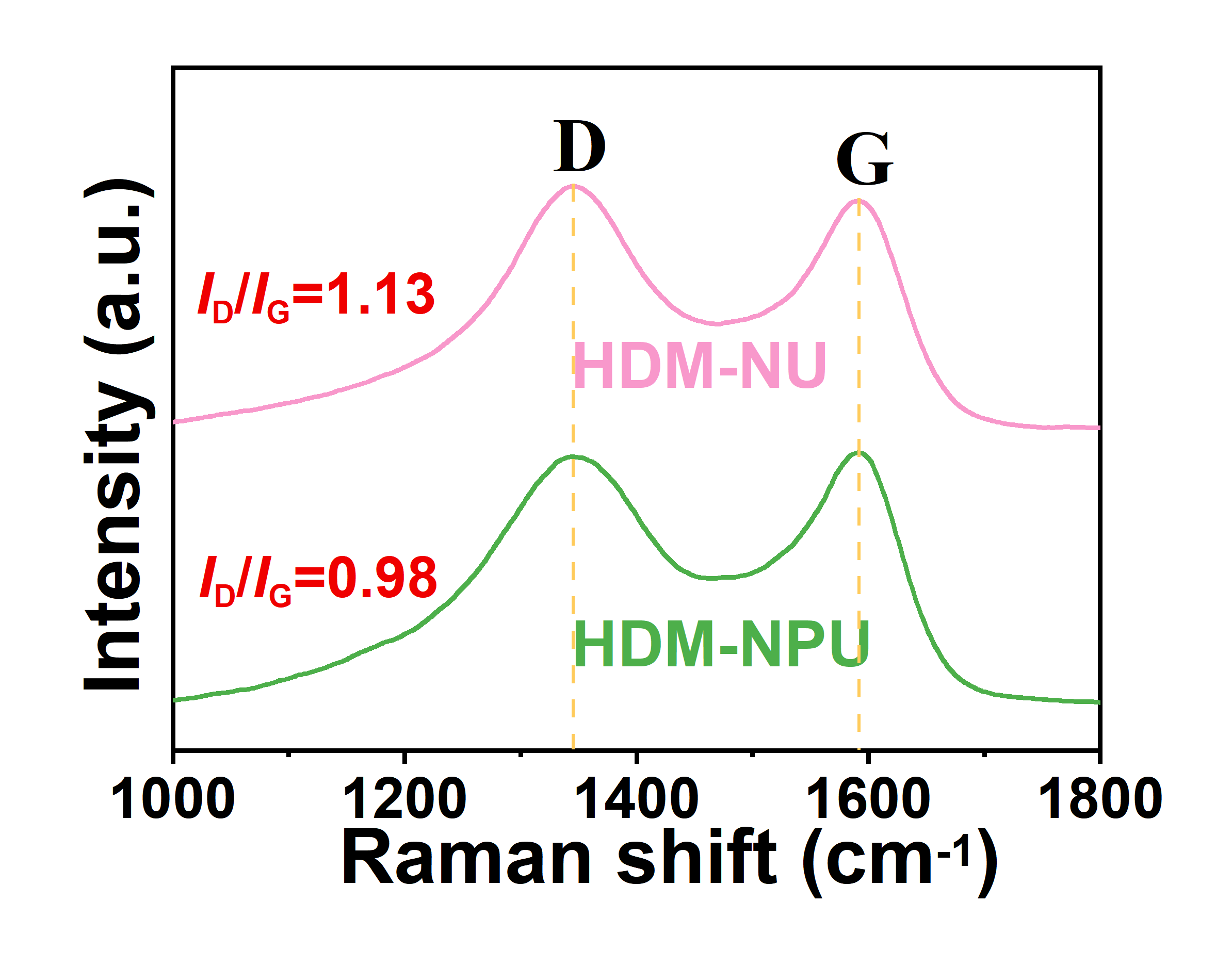


**Figure S3.** Raman spectra of preoxidized and unpreoxidized SLS-derived carbon (HDM-NPU and HDM-NU).

**5. N_2_/CO_2_ adsorption‒desorption isotherms and pore characteristic parameters of preoxidized and unpreoxidized SLS-derived carbon**

**
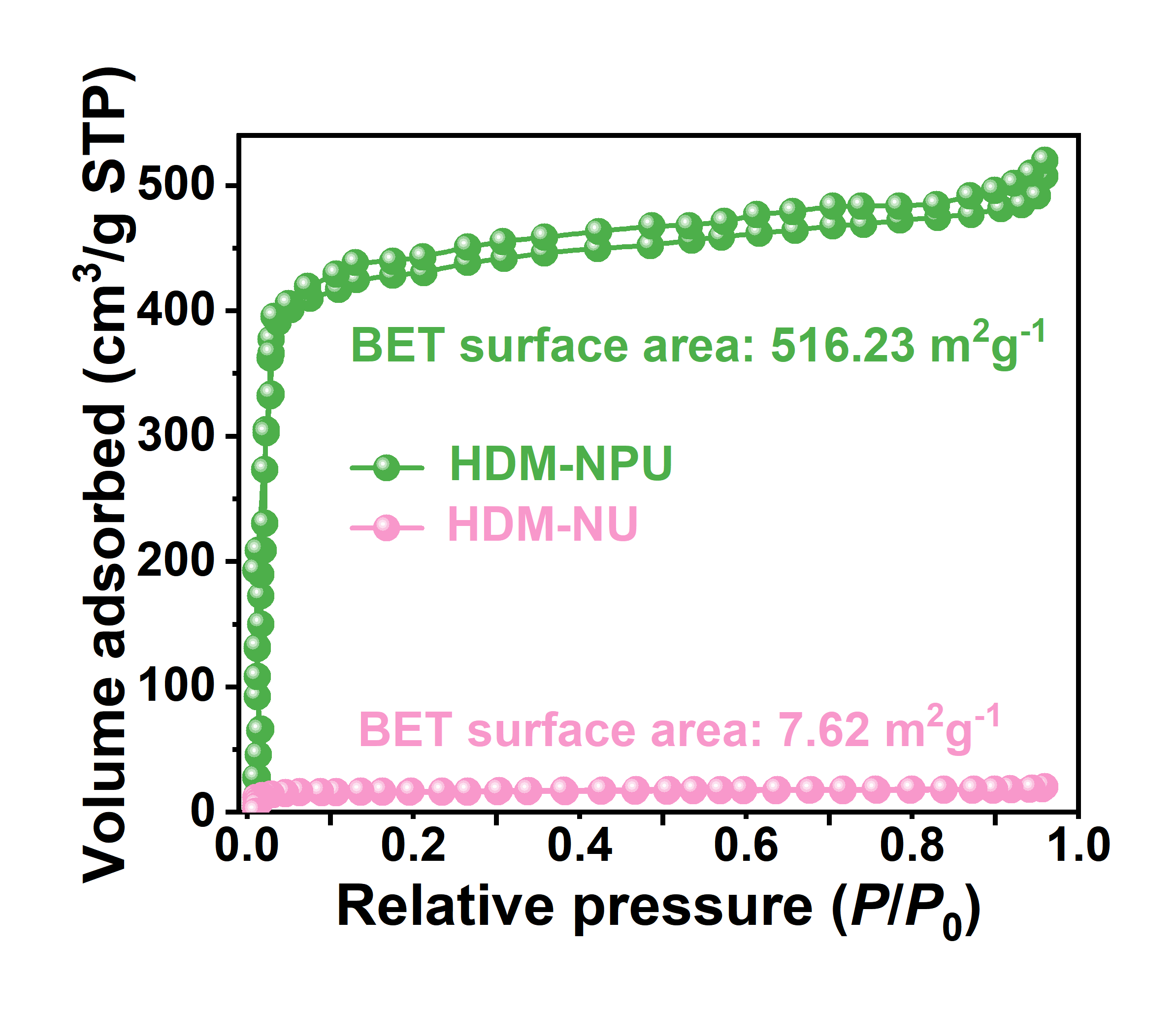
**

**Figure S4.** N_2_ adsorption‒desorption isotherms of preoxidized and unpreoxidized SLS-derived carbon (HDM-NPU and HDM-NU).


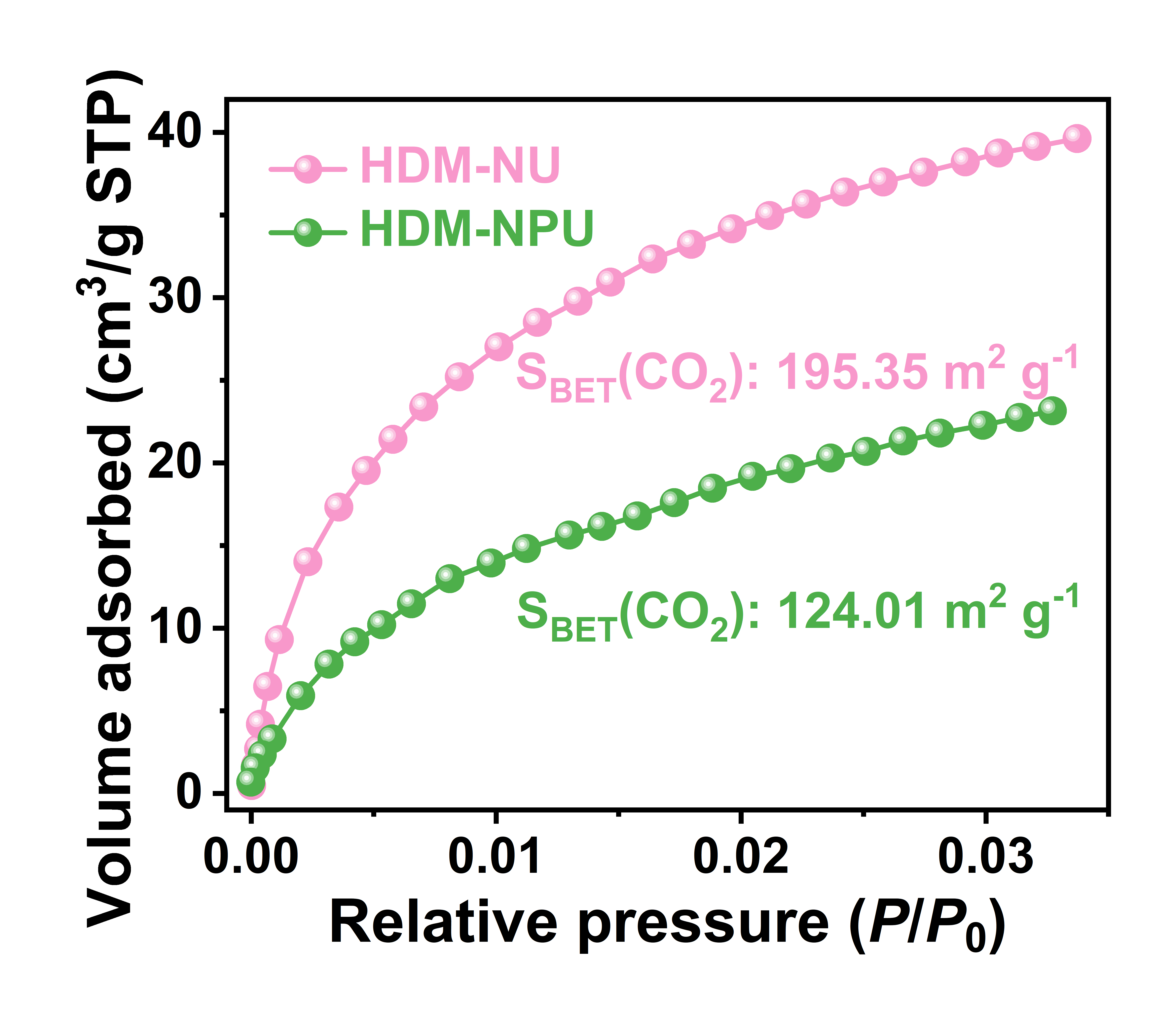


**Figure S5.** CO_2_ adsorption‒desorption isotherms of preoxidized and unpreoxidized SLS-derived carbon (HDM-NPU and HDM-NU).

**Table S2.** Pore characteristic parameters of preoxidized and unpreoxidized SLS-derived carbon (HDM-NPU and HDM-NU).

| **Samples** | *S*_BET_ (N_2_)  /m^2^ g^‒1^ | *V*_total_ (N_2_)  /cm^3^ g^‒1^ | *S*_BET_ (CO_2_)  /m^2^ g^‒1^ | *V*_total_ (CO_2_)  /cm^3^ g^‒1^ | Proportion of closed pores |
| --- | --- | --- | --- | --- | --- |
| **HDM-NPU** | 516.23 | 0.172 | 124.01 | 0.037 | 19.37% |
| **HDM-NU** | 7.62 | 0.003 | 195.35 | 0.068 | 96.25% |

**6. XPS survey spectra of N-S@HDM-1100, -1300, and -1500**

**
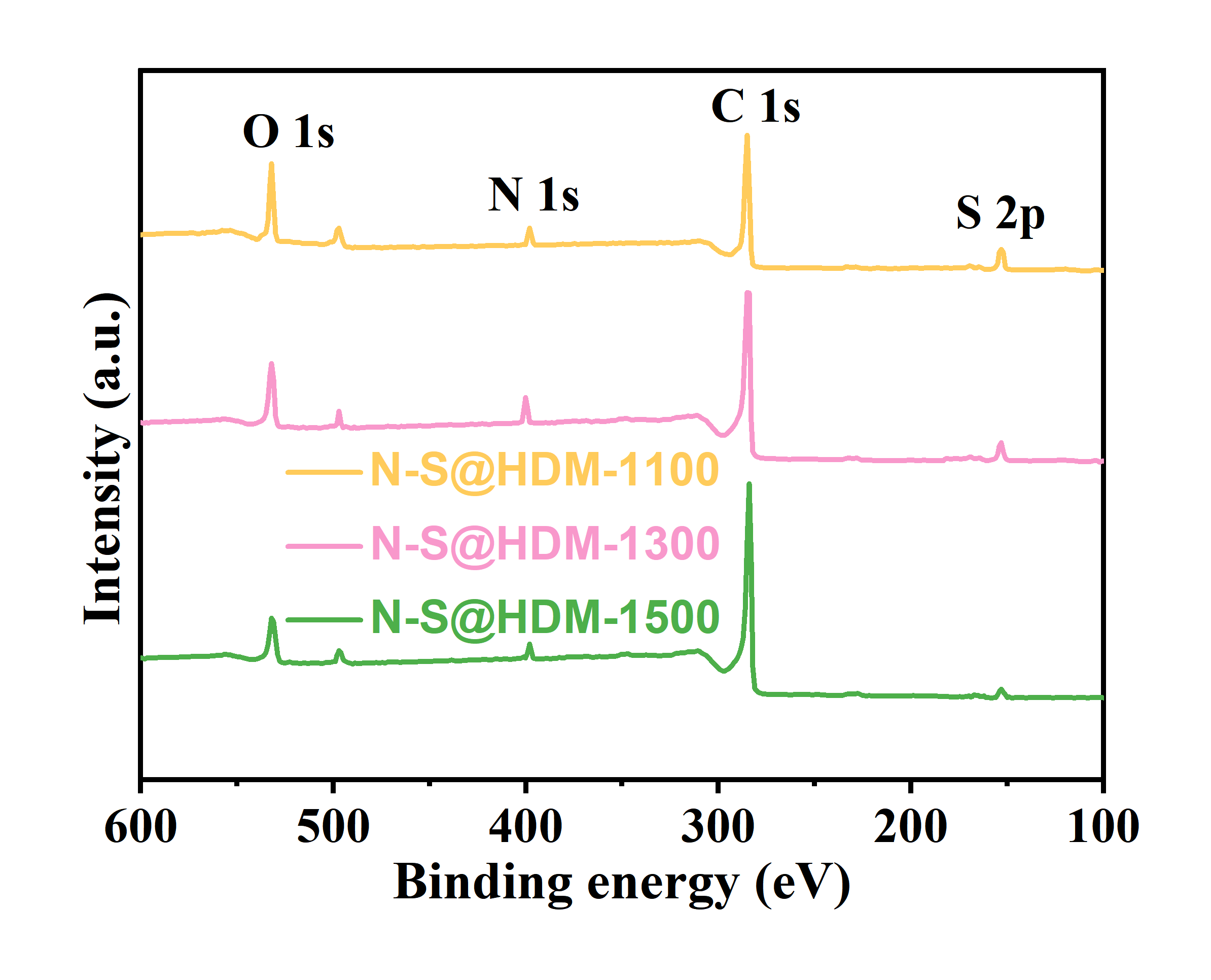
**

**Figure S6**. XPS survey spectra of N-S@HDM-1100, -1300, and -1500.

**7. Comparative analysis of deconvoluted peak proportions in the XPS C 1s, N 1s, and S 2p spectra of N-S@HDM-1100, -1300, and -1500**

**Table S3.** Comparative analysis of deconvoluted peak proportions in the XPS C 1s, N 1s, and S 2p spectra of N-S@HDM-1100, -1300, and -1500.

| **C 1s** | | | | | | | | |
| --- | --- | --- | --- | --- | --- | --- | --- | --- |
| **Samples** | C(O)O | | C=O | C**–**O | | C**–**C/C=O  /C**–**N | | C**–**S |
| **N-S@HDM-1100** | | 7.52%*^a^*  (11.13%)*^b^* | 10.91%  (16.04%) | | 14.28%  (20.92%) | 31.87%  (46.89%) | | 3.41%  (5.02%) |
| **N-S@HDM-1300** | | 5.23%  (8.02%) | 8.59%  (13.19%) | | 12.89%  (16.56%) | 41.08%  (55.14%) | | 4.89%  (7.09%) |
| **N-S@HDM-1500** | | 2.66%  (3.91%) | 4.22%  (5.21%) | | 6.79%  (9.61%) | 68.29%  (77.41%) | | 2.64%  (3.86%) |
| **N 1s** | | | | | | | | |
| **Samples** | | N–O | N–G | | N–5 | | N–6 | |
| **N-S@HDM-1100** | | 1.17%  (13.17%) | 2.14%  (19.54%) | | 2.66%  (33.04%) | | 2.94%  (34.25%) | |
| **N-S@HDM-1300** | | 1.69%  (15.02%) | 2.59%  (23.12%) | | 3.49%  (31.09%) | | 3.45%  (30.77%) | |
| **N-S@HDM-1500** | | 0.89%  (16.15%) | 1.80%  (30.52%) | | 1.64%  (27.41%) | | 1.73%  (25.92%) | |
| **S 2p** | | | | | | | | |
| **Samples** | | **–**SO_n_^–^ | | | C–S | | | |
| **N-S@HDM-1100** | | 6.85%  (66.34%) | | | 3.47%  (33.66%) | | | |
| **N-S@HDM-1300** | | 3.87%  (45.38%) | | | 4.66%  (54.62%) | | | |
| **N-S@HDM-1500** | | 1.61%  (35.15%) | | | 2.97%  (64.85%) | | | |

*^a^*The values outside the parentheses represent the actual content.

*^b^*The values inside the parentheses represent the relative content.

**8.** **Four-probe tests of electrode conductivity in N-S@HDM-1100, -1300, and -1500**


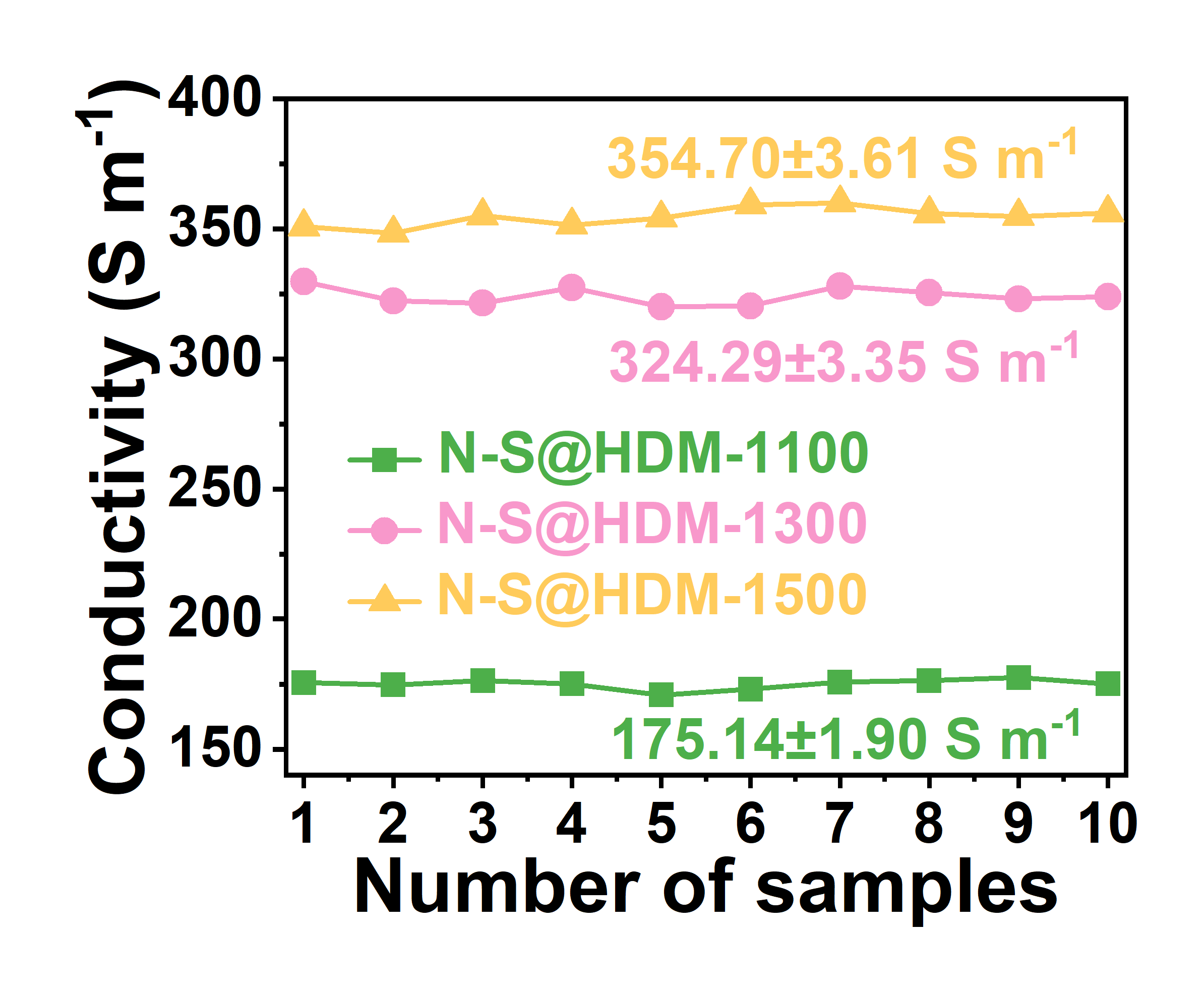


**Figure S7.** Four-probe tests of electrode conductivity in N-S@HDM-1100, -1300, and -1500.

**9.** **Four-probe tests of electrode conductivity in N-S@HDM-1300 and HDM-NPU**


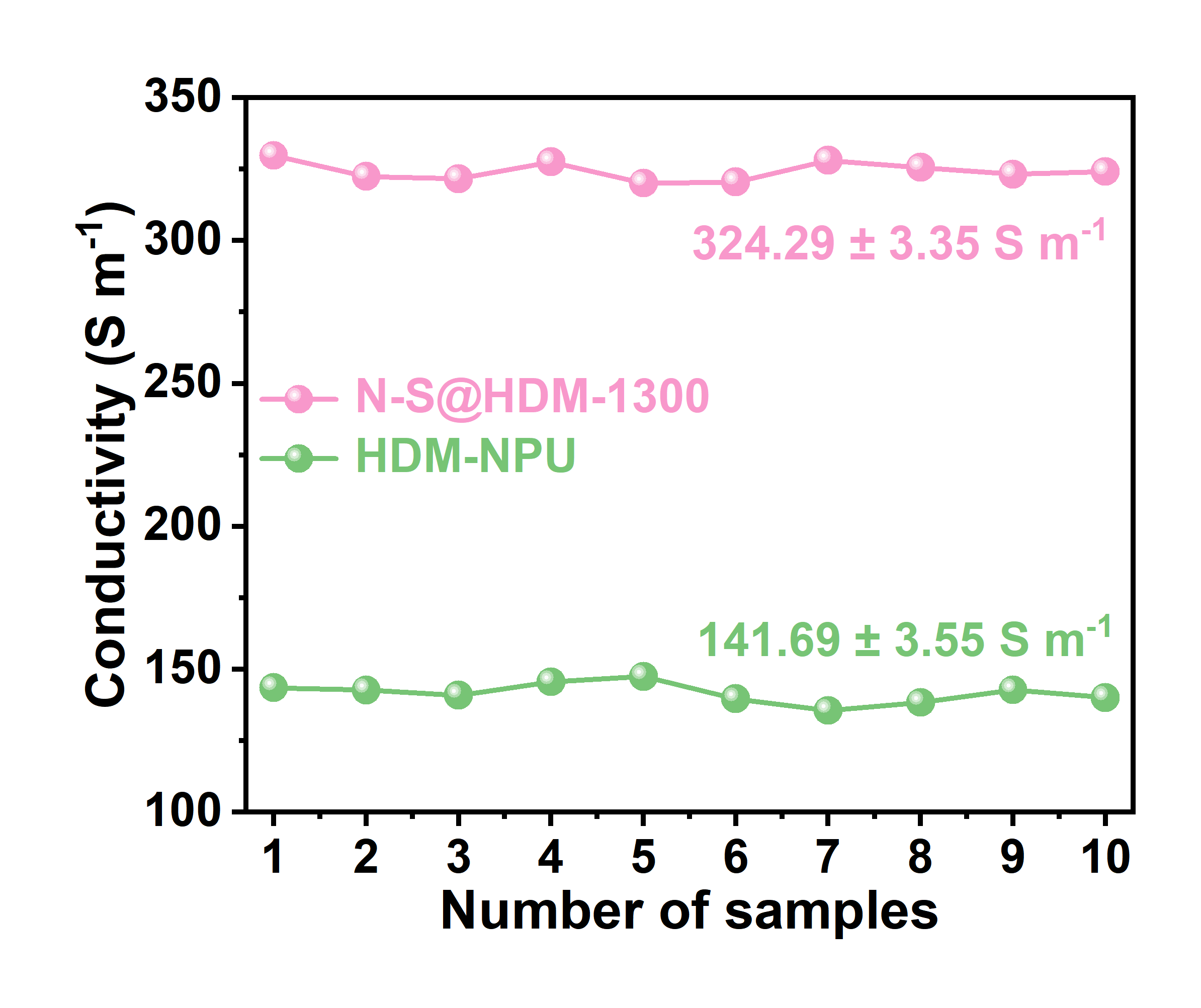


**Figure S8**. Four-probe tests of electrode conductivity in N-S@HDM-1300 and HDM-NPU.

**10. Tensile stress‒strain curves of N-S@HDM-1100, -1300, and -1500**


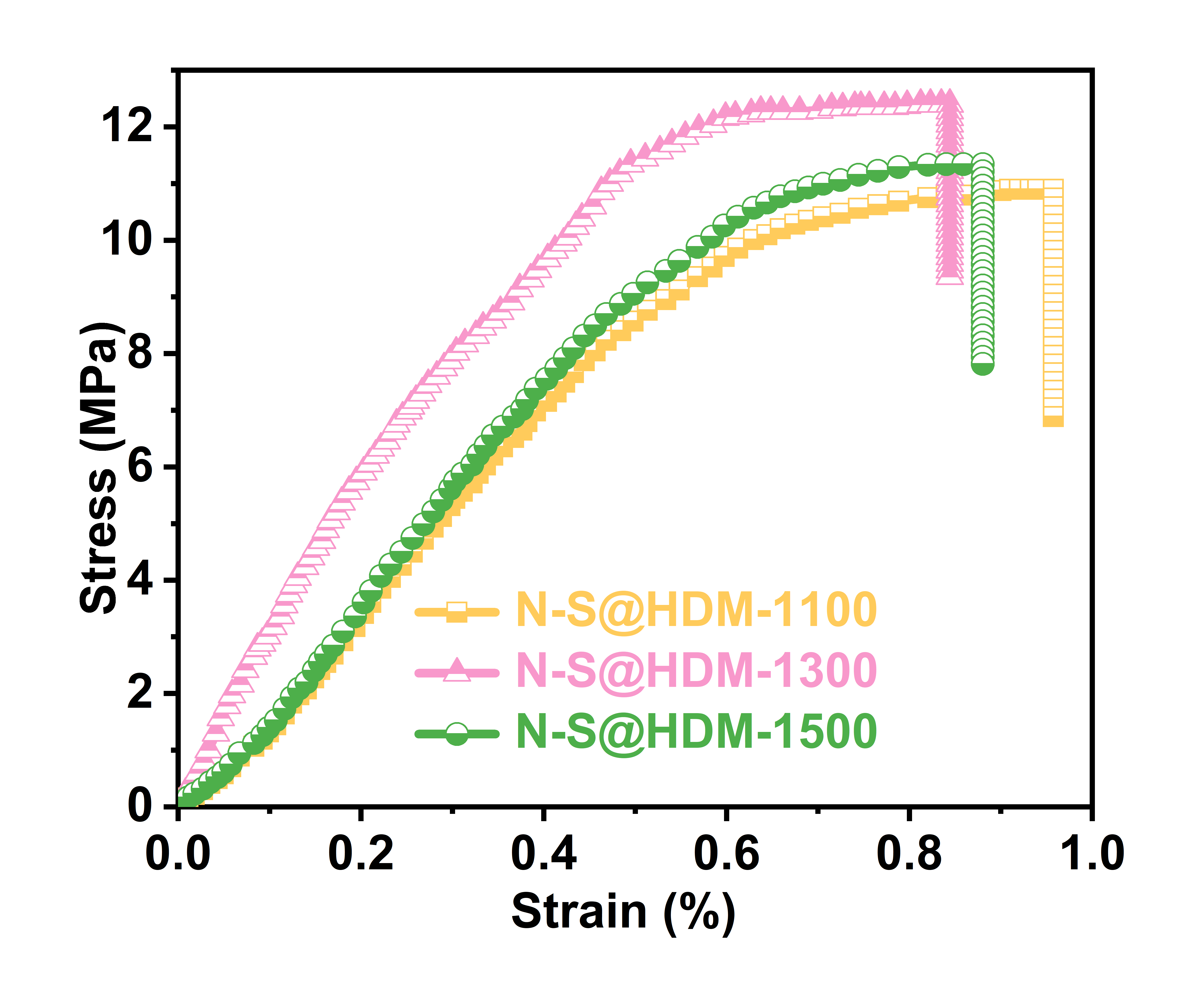


**Figure S9.** Tensile stress‒strain curves of N-S@HDM-1100, -1300, and -1500.

**11.** **Elemental contents of N-S@HDM-1100, -1300, and -1500**

**Table S4.** Elemental contents of N-S@HDM-1100, -1300, and -1500.

| **Samples** | C | N | O | S | Total doping ratio of N and S |
| --- | --- | --- | --- | --- | --- |
| **N-S@HDM-1100** | 67.99% | 8.91% | 12.77% | 10.33% | 19.24% |
| **N-S@HDM-1300** | 72.68% | 11.22% | 7.57% | 8.53% | 19.75% |
| **N-S@HDM-1500** | 84.60% | 6.06% | 4.76% | 4.58% | 10.64% |

**12.** **Calculation of crystalline size (*D*) and interplanar spacing (*d*_002_)**

The Scherrer equation establishes a correlation between the crystallite size and the full width at half maximum (FWHM) of a specific diffraction peak, measured at its peak intensity [1]:

$D=\frac{K\lambda}{\beta\cos\theta}$ (S1)

where *D* is the size perpendicular to the lattice plane, *K* is a constant and usually taken as 0.9, *λ* is the wavelength in the diffraction experiment, *β* is the PWHM, and ***θ*** is the diffraction angle.

The interplanar spacing (*d*_002_) is calculated from the Bragg equation [2]:

$d_{002}=\frac{\lambda}{2\sin\theta}$ (S2)

where *λ* is the wavelength of the incident X-rays, *d*_002_ is the interplanar spacing of the crystal, and *θ* is the Bragg's angle (°).


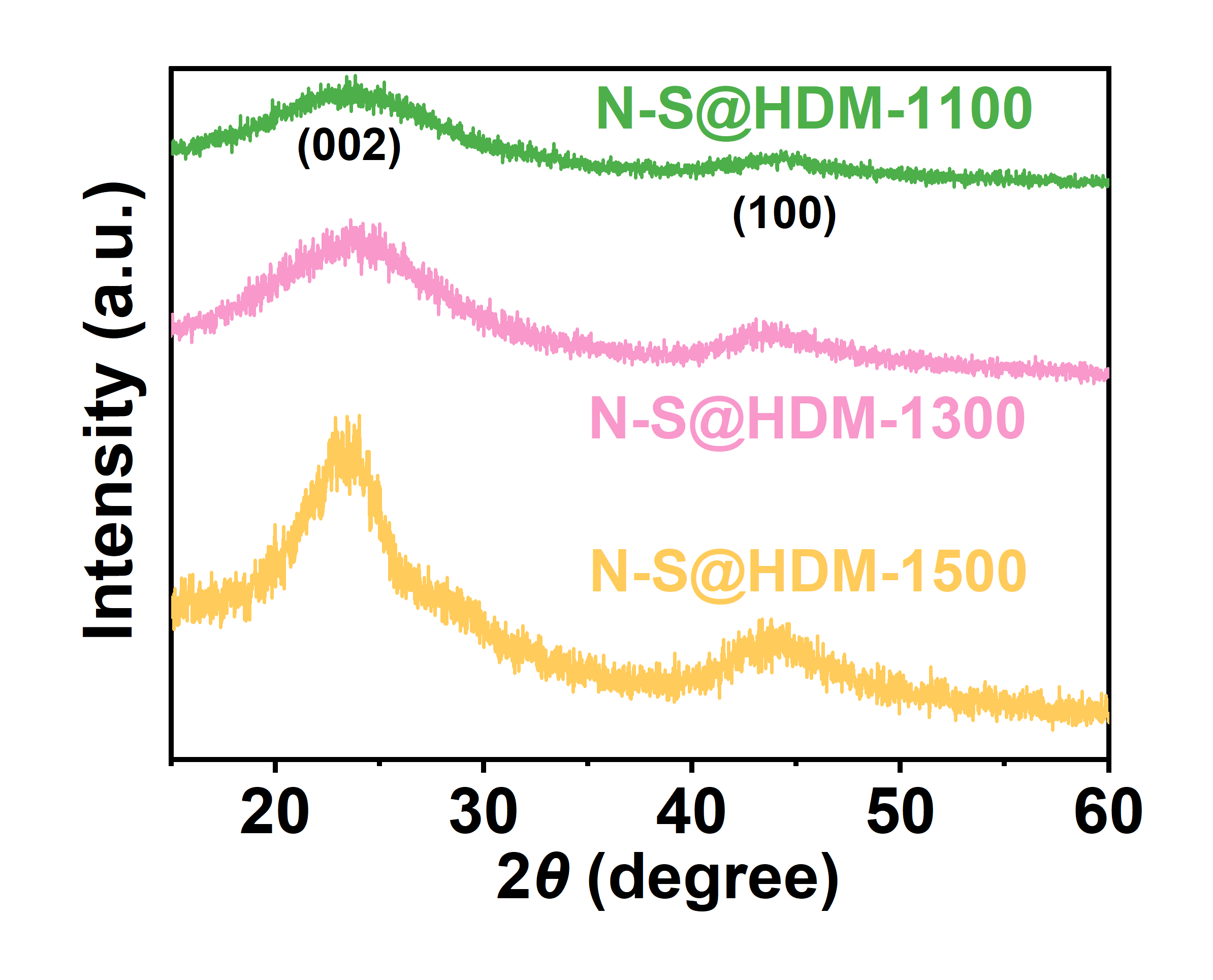


**Figure S10.** XRD patterns of N-S@HDM-1100, -1300, and -1500.

**Table S5.** FWHM, *θ,* crystallite size *D*, and *d*_002_ of N-S@HDM-1100, -1300, and -1500.

| **Samples** | 2*θ* (°) | FWHM | *D* (nm) | *d*_002_ (nm) |
| --- | --- | --- | --- | --- |
| **N-S@HDM-1100** | 22.7 | 9.7 | 0.84 | 0.399 |
| **N-S@HDM-1300** | 23.4 | 8.2 | 1.01 | 0.389 |
| **N-S@HDM-1500** | 24.2 | 4.3 | 1.93 | 0.375 |

**13.** **Formation mechanism of closed ultramicropores**


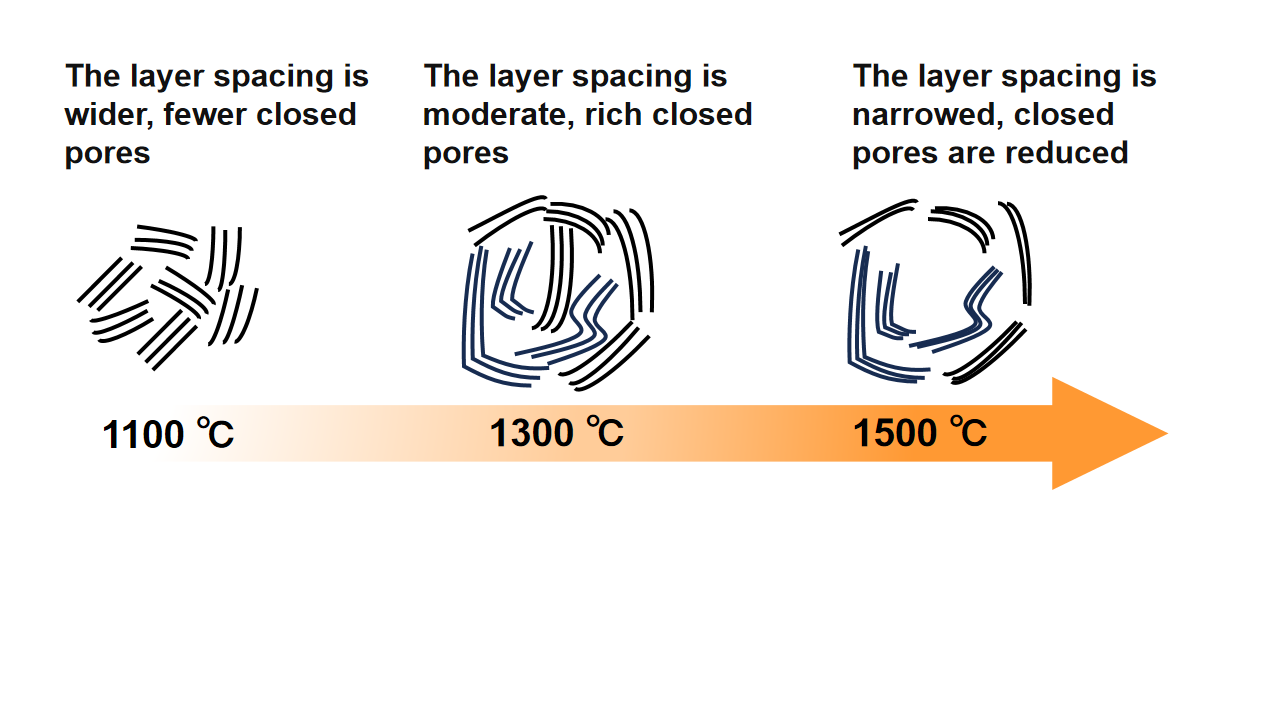


**Figure S11.** Schematic diagram illustrating the formation mechanism of closed ultramicropores based on the HRTEM observation.

**14.** **Raman spectra of N-S@HDM-1100, -1300, and -1500**

**
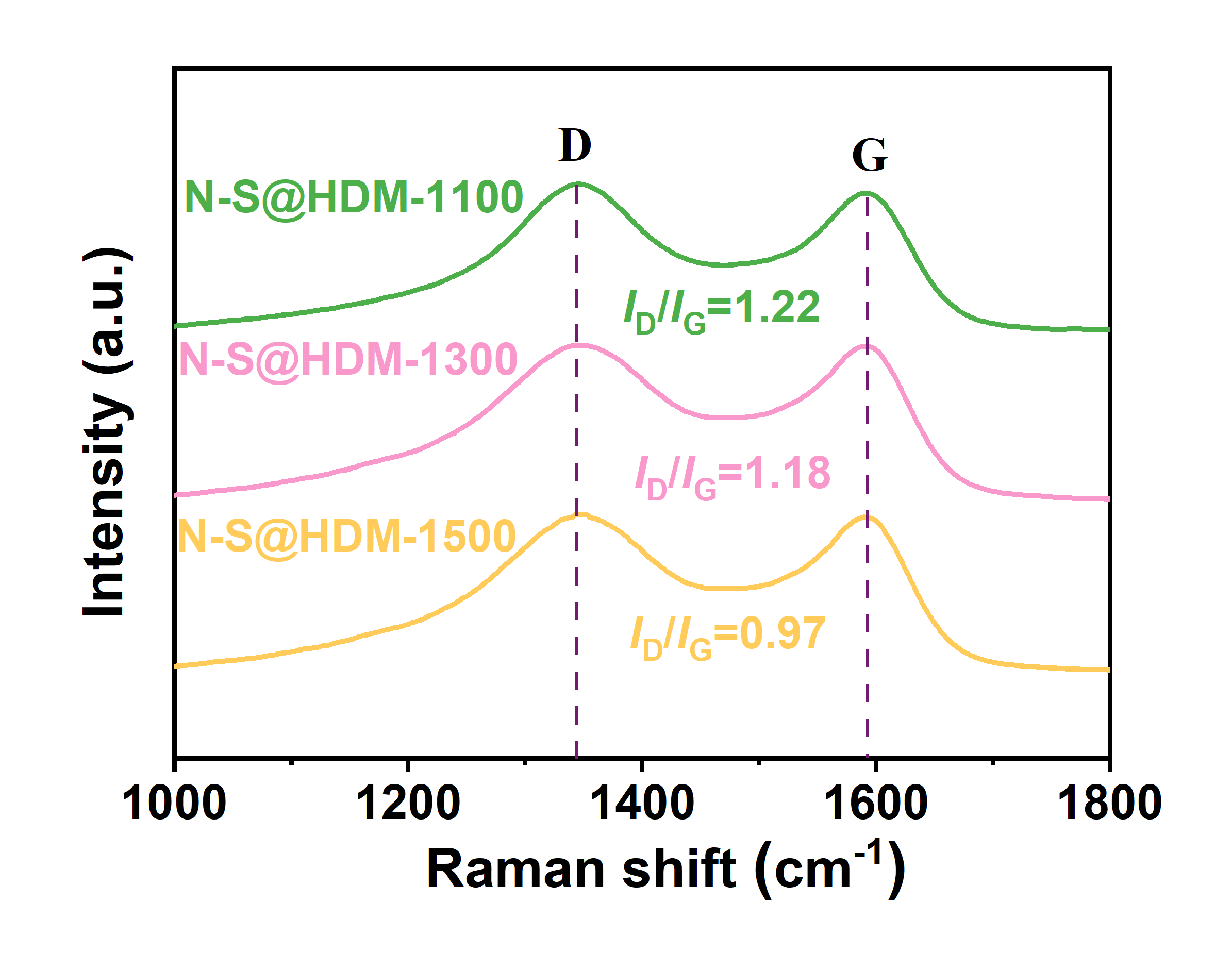
**

**Figure S12.** Raman spectra of N-S@HDM-1100, -1300, and -1500.

**15.** **N_2_ adsorption‒desorption isotherms of N-S@HDM-1100, -1300, and -1500**


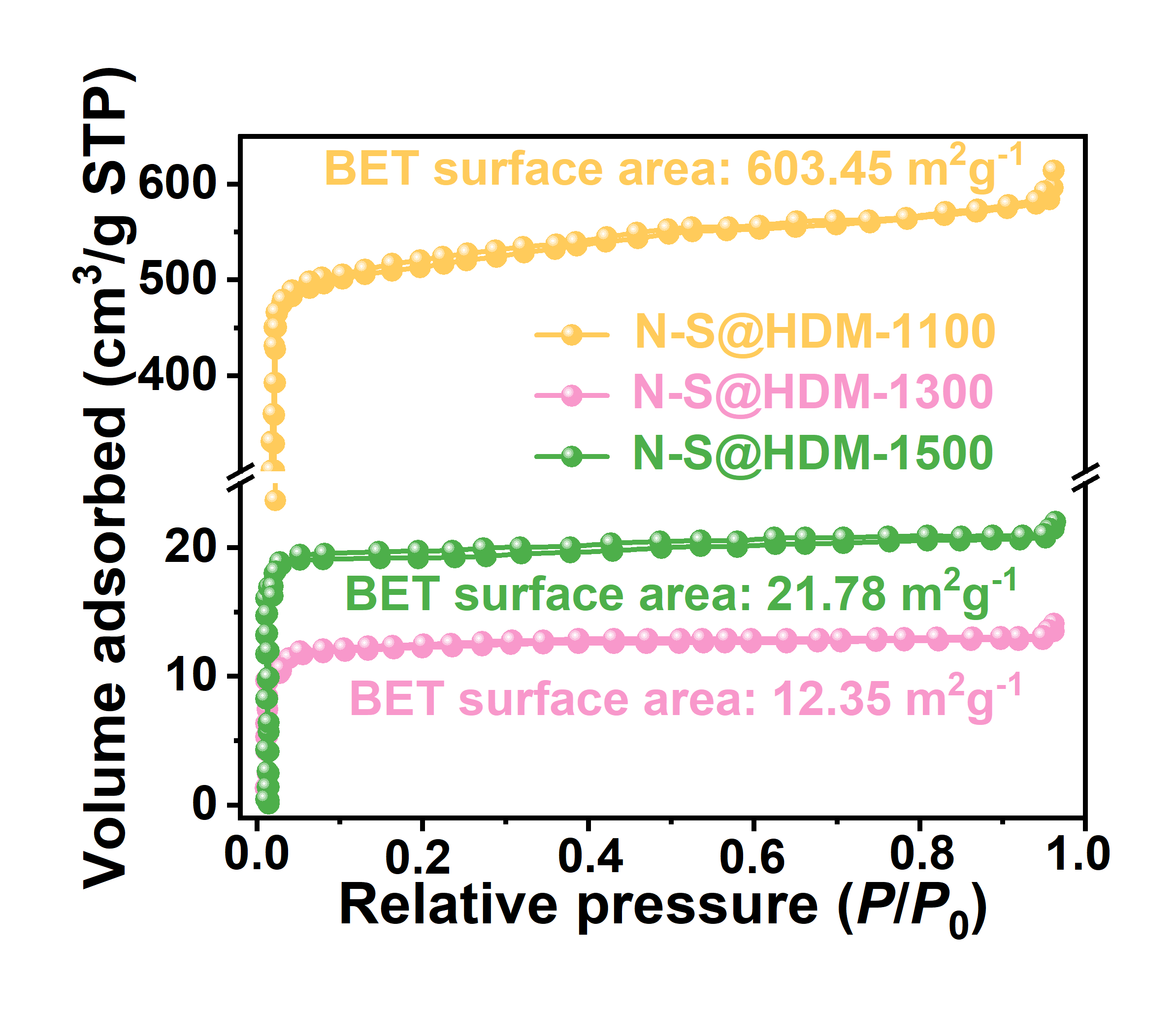


**Figure S13.** N_2_ adsorption‒desorption isotherms of N-S@HDM-1100, -1300, and -1500.

**16.** **Pore characteristic parameters of N-S@HDM-1100, -1300, and -1500**

**Table S6.** Pore characteristic parameters of N-S@HDM-1100, -1300, and -1500.

| **Samples** | *S*_BET_ (N_2_)  /m^2^ g^‒1^ | *V*_total_ (N_2_)  /cm^3^ g^‒1^ | *S*_BET_ (CO_2_)  /m^2^ g^‒1^ | *V*_total_ (CO_2_)  /cm^3^ g^‒1^ | Proportion of closed pores | *f*_a_ (SAXS) |
| --- | --- | --- | --- | --- | --- | --- |
| **N-S@HDM-1100** | 603.45 | 0.207 | 70.21 | 0.024 | 10.39% | 0.51 |
| **N-S@HDM-1300** | 12.35 | 0.004 | 203.23 | 0.071 | 94.27% | 0.85 |
| **N-S@HDM-1500** | 21.78 | 0.006 | 131.45 | 0.039 | 85.78% | 0.69 |

**17. An approach for fitting *f*_a_ in SAXS patterns utilizing the Teubner-Strey model**

SAXS measurements were performed using a Xeuss 2.0 system equipped with Cu-Kα radiation (*λ* = 1.542 Å) in transmission mode with parallel-beam geometry and a point detector. The experiment quantifies the scattering intensity *I*(Q) as a function of the scattering vector *Q*, where *Q* is defined by the equation:

$Q=\frac{4\text{π}}{\lambda}\sin\theta$ (S3)

where *λ* is the X-ray wavelength, which is *θ* the scattering angle.

The middle-angle scattering regime (*Q* = 0.1–1 Å^‒1^) of the SAXS profile was analyzed through Teubner-Strey model fitting [3,4], a methodology particularly suited for porous systems where interconnected networks exhibit short-range periodic order coexisting with long-range structural disorder, as exemplified by partially graphitized domains in hard carbon materials. This model uses three fitting parameters to describe the observed broad scattering peak, and the function takes the form:

$I(Q)=\frac{1}{A+BQ^{2}+CQ^{4}}$ (S4)

where the constraints that *A*, *B*, and *C* are variables which can be obtained by nonlinear least-squares fitting of the experimental data. This model enables the extraction of pore connectivity metrics, particularly through the amphiphilic factor (*f*_a_), which quantifies the interplay between short-range order and long-range disorder in porous networks. Specifically, *f*_a_ is derived from the model’s fitting parameters *A, B,* and *C*, which is calculated by the equation:

$f_{a}=\frac{B}{\sqrt{4AC}}$ (S5)

**18. Ture density and porosity of N-S@HDM-1100, -1300, and -1500**

**Table S7.** Ture density and porosity of N-S@HDM-1100, -1300, and -1500.

| **Samples** | **Ture density/(g cm^‒3^)** | **Porosity/%** |
| --- | --- | --- |
| **N-S@HDM-1100** | 2.06 | 78.97 |
| **N-S@HDM-1300** | 1.72 | 89.25 |
| **N-S@HDM-1500** | 2.01 | 79.06 |

**19. Electrochemical properties of N-S@HDM-1300, HDM-NPU, HDM-NU, and HDM-NP**


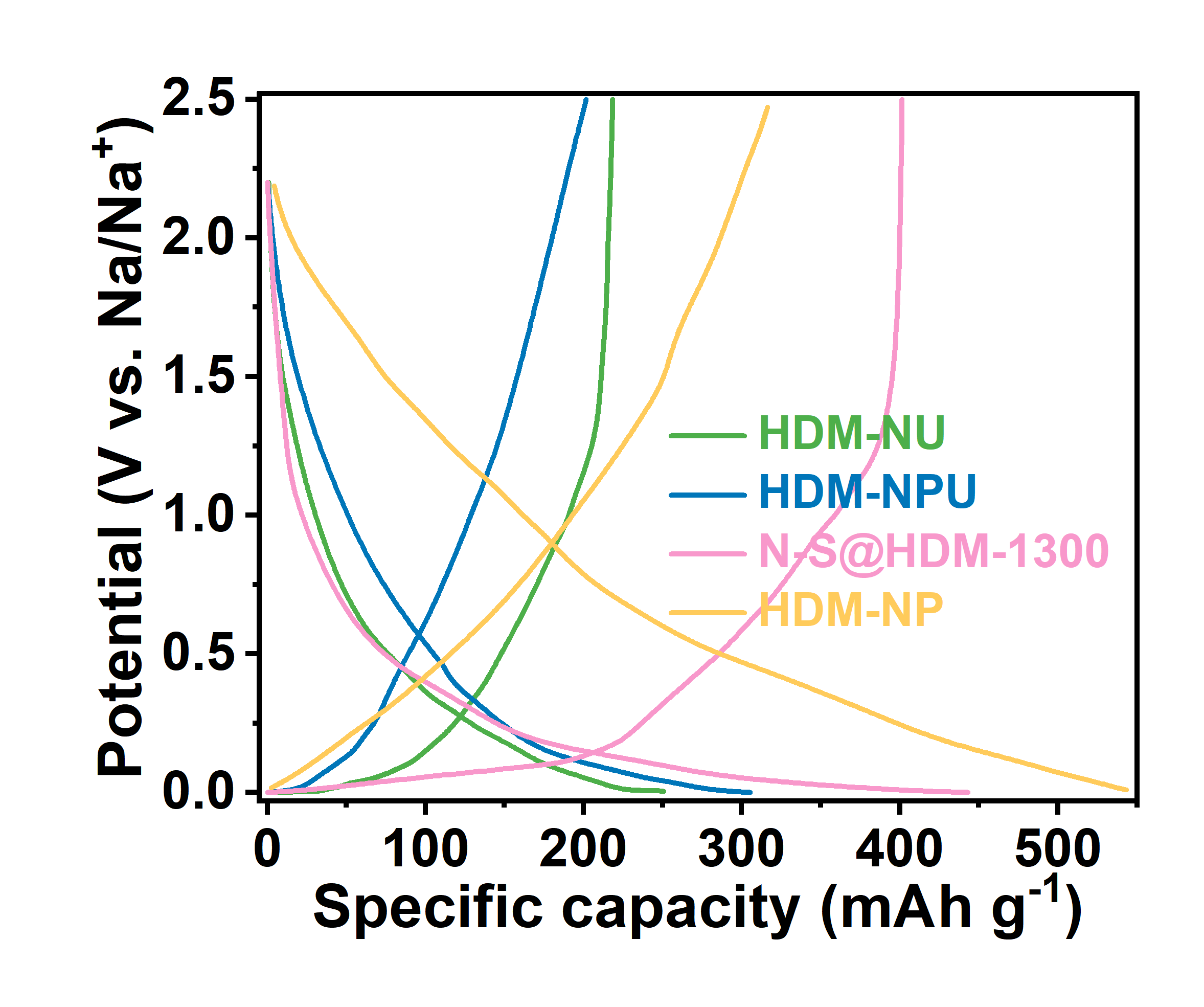


**Figure S14**. Initial GCD profiles of N-S@HDM-1300, HDM-NPU, HDM-NU, and HDM-NP at 30 mA g^–1^.

**Table S8.** Summary of electrochemical properties of N-S@HDM-1300, HDM-NPU, HDM-NU, and HDM-NP tested at 30 mA g^–1^.

| **Samples** | ICE | Specific capacity (mAh g^–1^) |
| --- | --- | --- |
| **HDM-NPU** | 66.1% | 201.5 |
| **HDM-NU** | 87.2% | 218.6 |
| **HDM-NP** | 58.2% | 316.4 |
| **N-S@HDM-1300** | 90.6% | 401.5 |

**20. XPS survey spectra of SLS, POSLS, N-S@HDM-1300, HDM-NPU, HDM-NU, and HDM-NP**


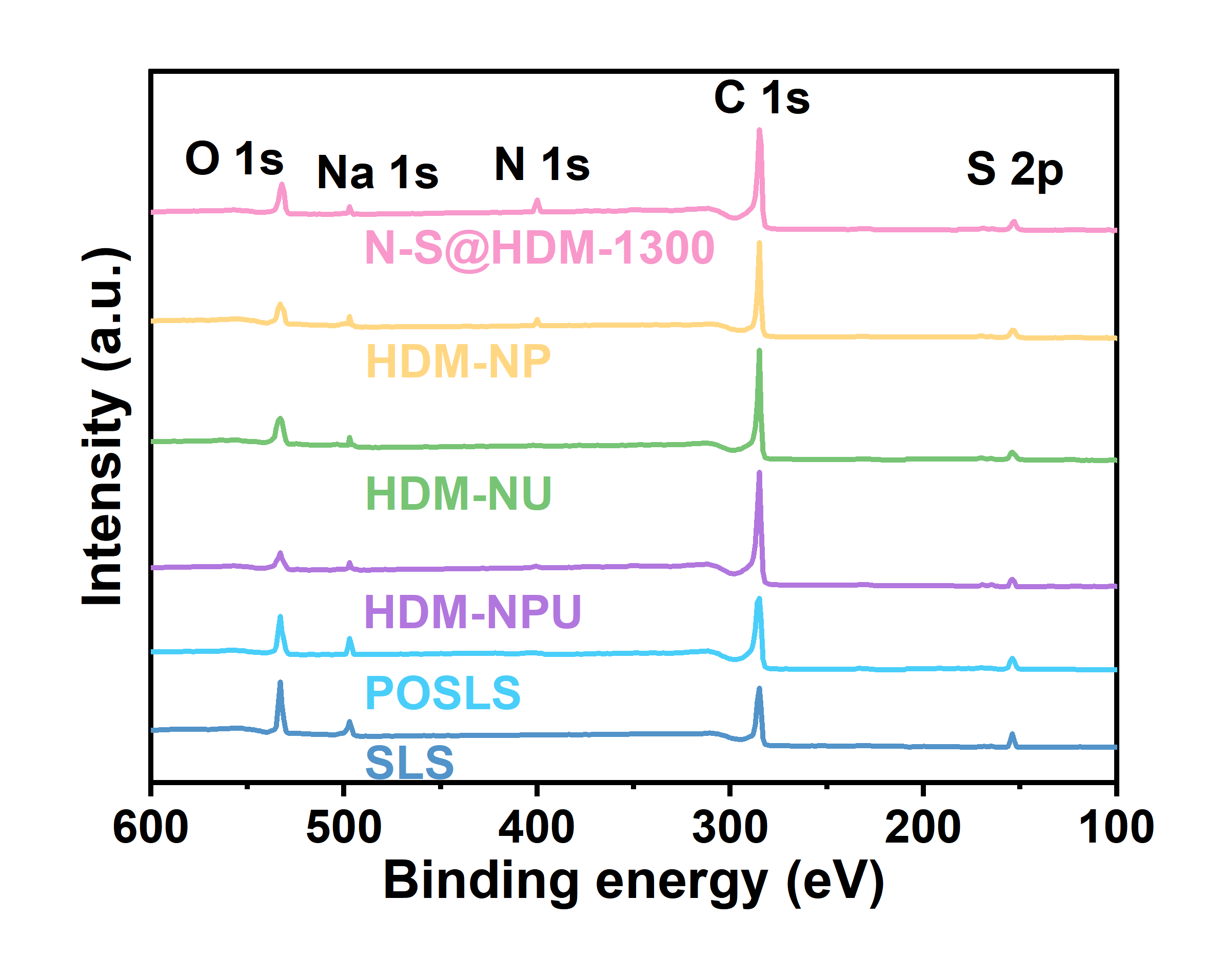


**Figure S15**. XPS survey spectra of SLS, POSLS, N-S@HDM, HDM-NPU, HDM-NU, and HDM-NP.

**Table S9.** Elemental contents of SLS, POSLS, N-S@HDM, HDM-NPU, HDM-NU, and HDM-NP.

| **Samples** | C (%) | N (%) | O (%) | S (%) | Na (%) |
| --- | --- | --- | --- | --- | --- |
| **N-S@HDM-1300** | 70.08 | 11.03 | 7.26 | 8.42 | 3.21 |
| **HDM-NP** | 73.59 | 10.04 | 4.36 | 8.15 | 3.86 |
| **HDM-NU** | 81.07 | 0 | 7.04 | 8.12 | 3.77 |
| **HDM-NPU** | 84.10 | 0 | 4.22 | 8.03 | 3.65 |
| **POSLS** | 57.09 | 0 | 22.13 | 11.16 | 9.62 |
| **SLS** | 48.95 | 0 | 28.59 | 13.35 | 9.11 |

**21. Evidences for the existence of sodium clusters**

To demonstrate the existence of sodium clusters, specifically, we prepared and analyzed two samples:

(1) N-S@HDM-1300 (0 cycle): A freshly prepared electrode before cycling;

(2) N-S@HDM-1300 (10 cycle): An electrode that was discharged to 0.01 V (fully sodiated) after 10 stable GCD cycles, then carefully disassembled, washed, and dried to preserve its sodiated state.


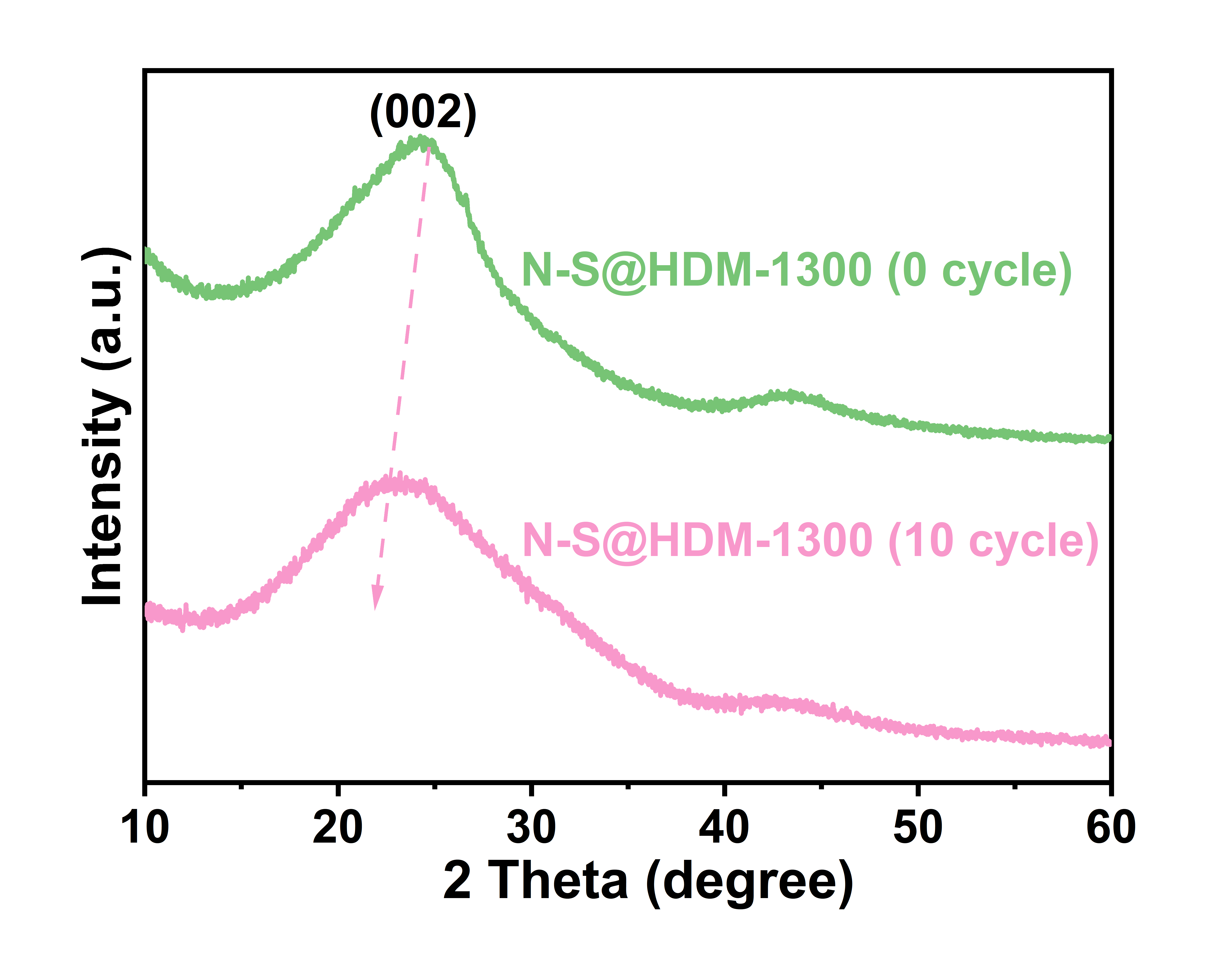


**Figure S16**. XRD patterns of N-S@HDM-1300 (0 cycle) and N-S@HDM-1300 (10 cycle).


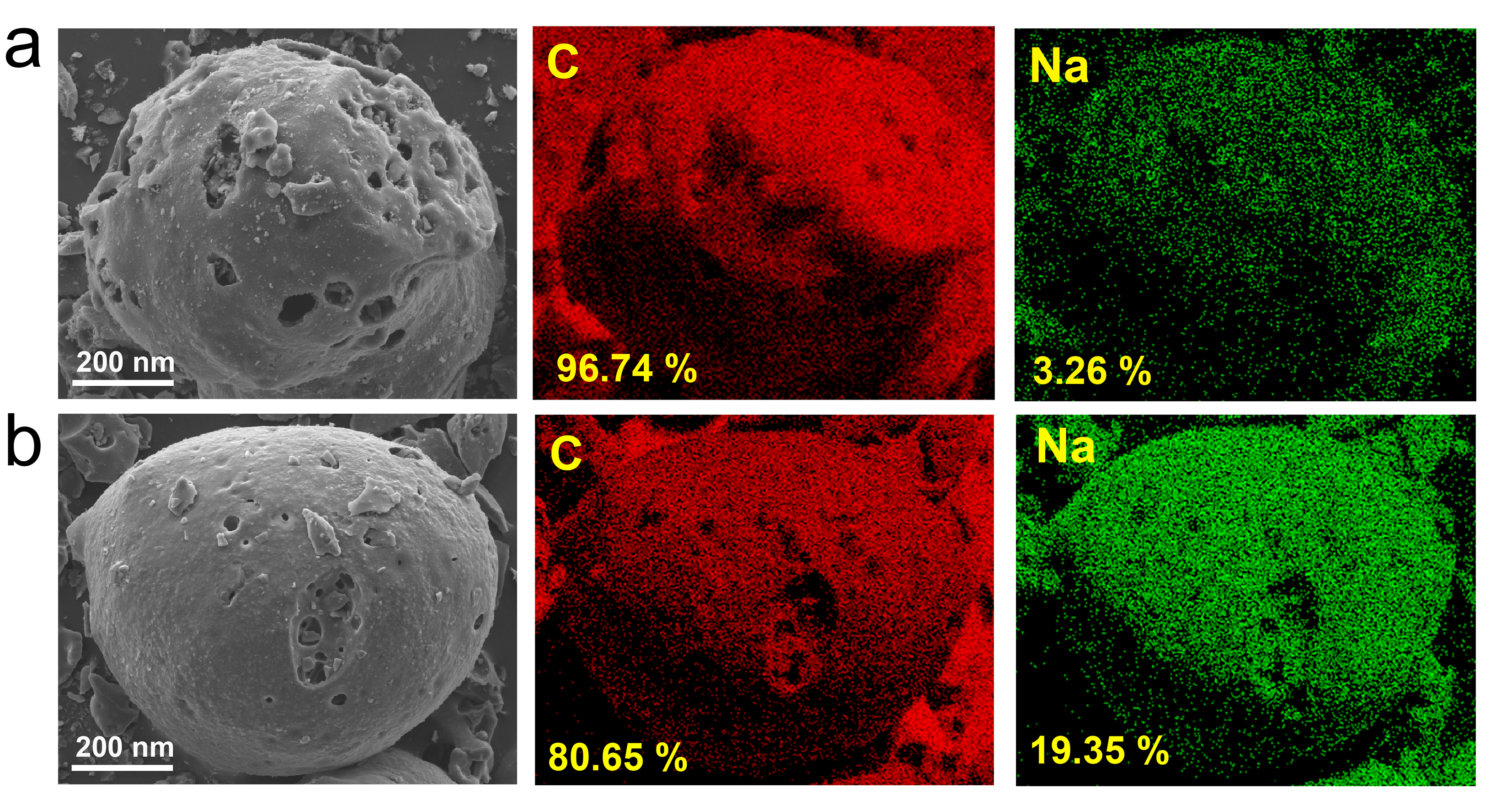


**Figure S17.** SEM images and EDX elemental mapping comparing the (a) pristine (0 cycle) and (b) cycled (10 cycles) electrodes.

**22. Fitted parameters of the Randles equivalent circuit for Nyquist plots**

**Table S10.** Fitted parameters of the Randles equivalent circuit for HDM-NPU and N-S@HDM-1100, -1300, and -1500.

| Samples | *R*_s_ (Ω) | *R*_ct_ (Ω) | *Z*_W_ (Ω) | CPE-P*^a^* | CPE-n*^b^* |
| --- | --- | --- | --- | --- | --- |
| HDM-NPU | 7.4 | 160.5 | 165.8 | 0.00028 | 0.71 |
| N-S@HDM-1100 | 6.8 | 133.8 | 141.2 | 0.00034 | 0.67 |
| N-S@HDM-1300 | 4.4 | 74.8 | 85.6 | 0.00049 | 0.56 |
| N-S@HDM-1500 | 5.6 | 75.9 | 87.3 | 0.00041 | 0.61 |

***^a^***P represents the pre-exponential factor of CPE; ***^b^***n is the exponent of CPE.

**23. A summary of electrochemical properties for HDM-NPU, N-S@HDM-1100, -1300, and -1500**

**Table S11.** Electrochemical properties of HDM-NPU, as well as N-S@HDM-1100, -1300, and -1500.

| **Sample** | ICE | Specific capacity  (mAh g^-1^) | Cyclic performance | Rate performance*^a^* |
| --- | --- | --- | --- | --- |
| **HDM-NPU** | 66.1% | 201.5 | 70.9% | 19.8% |
| **N-S@HDM-1100** | 60.7% | 234.8 | 72.0% | 21.9% |
| **N-S@HDM-1300** | 90.6% | 401.5 | 95.0% | 68.7% |
| **N-S@HDM-1500** | 82.4% | 305.1 | 87.3% | 25.6% |

*^a^*The rate performance is quantified by taking the ratio of the specific capacity measured at 5000 mA g^‒1^ to that measured at 30 mA g^‒1^.

**24. Electrochemical performance comparison of SIBs using 18 biochar variants**

**Table S12.** Comparison of electrochemical properties between N-S@HDM-based SIBs and SIBs utilizing 17 kinds of biomass-derived carbons.

| **Precursors** | | **Synthesis strategy** | **Capacity/(mAh g^‒1^)** | **ICE/%** | **Cycle number** | **Capacity retention rate/%** | **Refs.** |
| --- | --- | --- | --- | --- | --- | --- | --- |
| **SLS** | **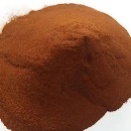** | **Synergistic ultramicropore-confined and electronic-state modulation strategies** | **401.5** | **90.6%** | **500** | **95.0** | **This work** |
| Sugarcane | 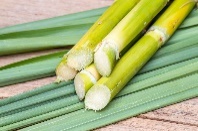 | Chemical treatment combined with carbonization | 229.0 ↓ | 78.2 ↓ | 50 ↓ | 82.5 ↓ | 5 |
| Lignin | 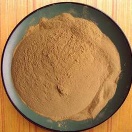 | Acetylation pretreatment and carbonization | 292.0 ↓ | 62.8 ↓ | 200 ↓ | 83.0 ↓ | 6 |
| Water-soluble starch  Bamboo | 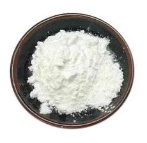  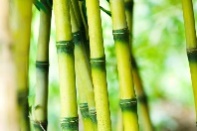 | Hydrothermal carbonization combined with chemical activation  Free radical regulation strategy | 343.1 ↓  350.0 ↓ | 80.1 ↓  86.8 ↑ | 200 ↓  100 ↓ | 87.2 ↓  96.0 ↑ | 7  8 |
| Cob | 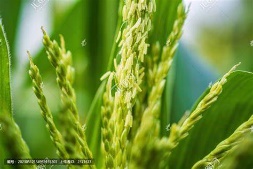 | Precarbonization, ball milling, and high-temperature pyrolysis | 311.3 ↓ | 79.6 ↓ | 100 ↓ | 80.4 ↓ | 9 |
| Garlic | 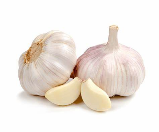 | High-temperature pyrolysis and S/N self-doping | 260.0 ↓ | 50.7 ↓ | 500 | 88.5 ↓ | 10 |
| Peanut shell | 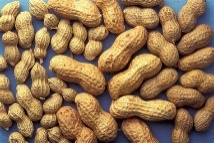 | Hydrothermal pretreatment combined with carbonization | 261.0 ↓ | 58.0 ↓ | 100 ↓ | 95.0 ↓ | 11 |
| Sucrose  Lotus stem  Recycled cork  Cotton  Kapok fiber  Reed straw  Bagasse  Sawdust  Waste tea  Cucumber stem | 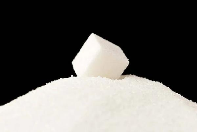  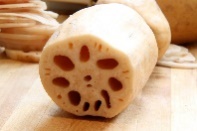  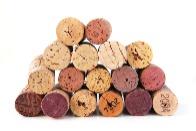  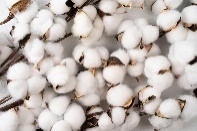  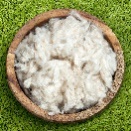  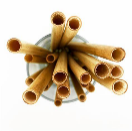  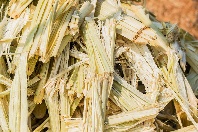  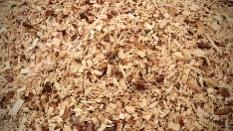  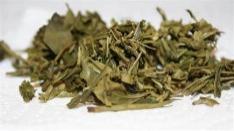  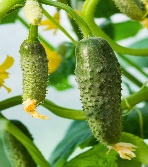 | Hydrothermal carbonization and vapor deposition  Direct carbonization  Precarbonization and high-temperature pyrolysis  Microtubule structure biomimetic strategy  Precarbonization strategy  Synergistic gradient temperature carbonization  High-temperature gradient thermal decomposition strategy  ZnO template-assisted carbonization synthesis  Acid pretreatment combined with carbonization  Controlled pyrolysis and KOH activation strategy | 300.0 ↓  351.0 ↓  385.0 ↑  315.0 ↓  290.0 ↓  372.0 ↓  242.1 ↓  275.0 ↓  282.4 ↓  337.9 ↓ | 83.0 ↓  70.0 ↓  81.0 ↓  73.0 ↓  80.0 ↓  77.1 ↓  73.1 ↓  56.0 ↓  69.0 ↓  64.9 ↓ | 100 ↓  450 ↓  500  100 ↓  200 ↓  200 ↓  500  500  200 ↓  500 | 93.0 ↓  94.0 ↓  81.6 ↓  92.0 ↓  93.0 ↓  84.0 ↓  93.4 ↓  69.8 ↓  83.0 ↓  82.1 ↓ | 12  13  14  15  16  17  18  19  20  21 |

**25. Electrochemical properties of commercial hard carbon**

**Table S13.** Electrochemical properties of commercial hard carbon.

| Sample | ICE (%) | Specific capacity (mAh g^-1^) | Sources |
| --- | --- | --- | --- |
| N-S@HDM-1300 | 90.6 | 401.5 | This work |
| T-Na-HC01 | --- | 250.0 | <https://www.tobmachine.com/irregular-hard-carbon-powder-materials-for-sodium-ion-and-lithium-ion-battery_p1013.html> |
| MSE PRO | 84.0 | 290.0 | <https://www.msesupplies.com/products/mse-pro-hard-carbon-powder-for-lithium-and-sodium-ion-battery-anode-100g?variant=31887616245818&utm_source=google&utm_medium=cpc&utm_campaign=20557420255&utm_term=&gad_source=1&gad_campaignid=20557421431&gclid=Cj0KCQjw9czHBhCyARIsAFZlN8Q4pozH9vpCiHBd1mHctLdGLiD3z18is1EmBlbNk5hwgkrtPiqmxQEaAiibEALw_wcB> |
| BSHC-300 BTR | 88.0 | 295.0 | https://www.btrchina.com/en/NegativeProducts/info.aspx?itemid=1063 |
| XW-C | 86.0 | 170.0 | <https://xwmaterial.com/product/sodium-ion-hard-carbon-powder/> |

**26.** **Operational endurance demonstration: comparative analysis with commercial AG625 batteries**

**Movie S1**. Demonstration of operational endurance for N-S@HDM-based half-cells powering a 15 W fan (10x normal speed).

**Movie S2**. Demonstration of operational endurance for commercial AG625 batteries powering a 15 W fan (10x normal speed).

**Please find these two movies attached.**

**27. Comparison of energy and power density of the assembled sodium-ion full-cell**

**Table S14.** Comparison of energy and power density of N-S@HDM-based full-cell against other recently reported full-cells.

| **Sample** | Energy density  (Wh kg^-1^) | Power density  (W kg^-1^) | Refs. |
| --- | --- | --- | --- |
| **2D layered structure MoTe_2_//NVP (coded as MoTe_2_//NVP)** | 110.1 | 29.2 | 22 |
| **MoSe_2_-Covered N/P-doped carbon nanosheets//NVP (coded as MoSe_2_//NVP)** | 143.2 | 101.4 | 23 |
| **Magnesium gluconate and glucose-based hard carbon//NNMTO (coded as HC//NNMTO)** | 199.7 | 15.1 | 24 |
| **Zinc-doped hard carbon//NVP (coded as ZnHC//NVP)** | 62.2 | 309.7 | 25 |
| **Anthracite flash Joule heating preparation of hard carbon//NVP (coded as FJH//NVP)** | 225.3 | 22.5 | 26 |
| **Rice husk-derived hard carbon//NVP (coded as RHHC-1300//NVP)** | 185.4 | 50.1 | 27 |
| **Regulating ternary graphite intercalation compounds//NVP (coded as graphite//NVP)** | 140.3 | 260.4 | 28 |
| **N-S@HDM-1300//NVP** | 215.5 | 36.0 | **This Work** |
|  | 172.4 | 118.1 | **This Work** |
|  | 148.6 | 235.3 | **This Work** |
|  | 130.1 | 348.2 | **This Work** |
|  | 106.5 | 578.3 | **This Work** |

**28. HRTEM images of the SEI film**


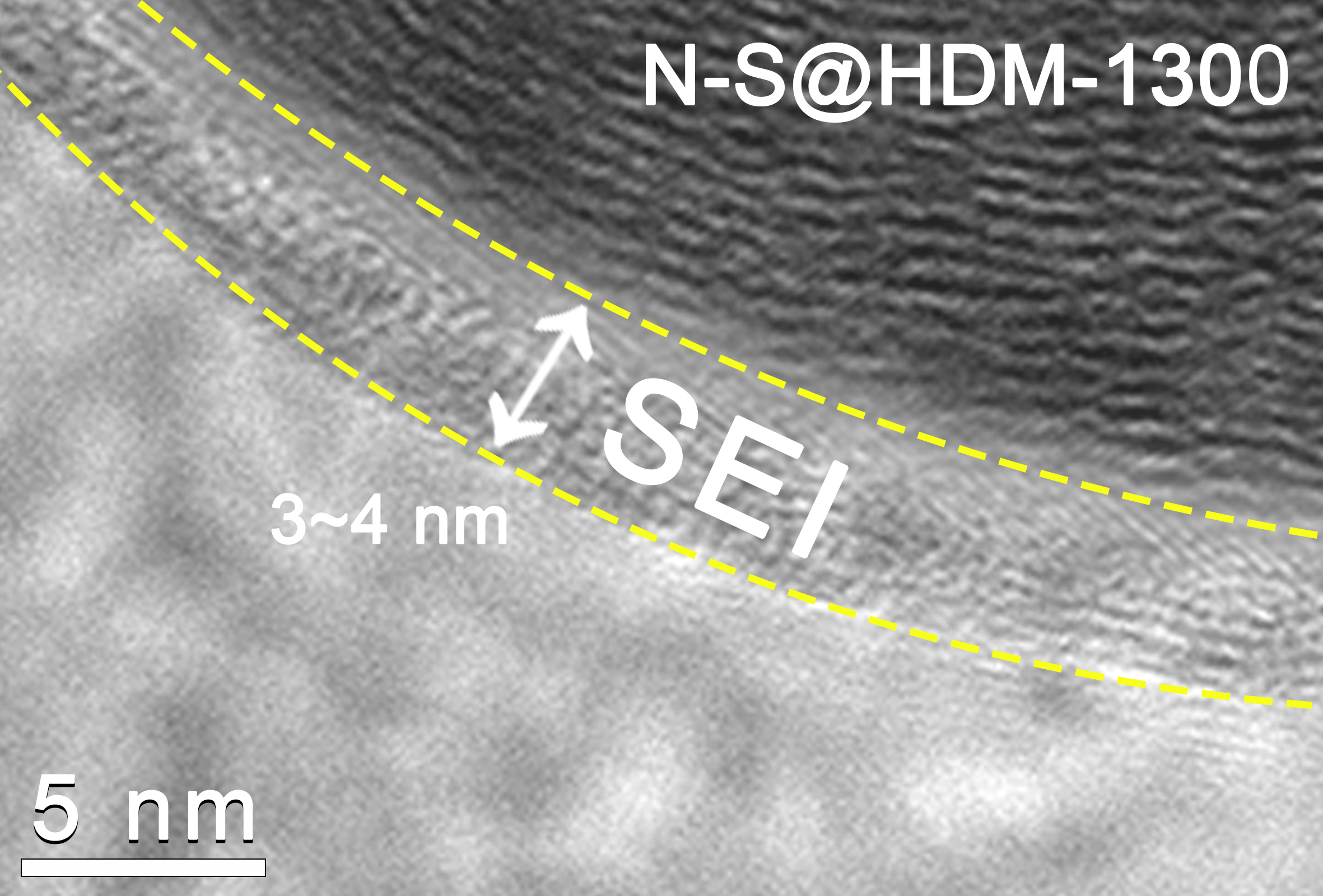


**Figure S18**. HRTEM images of the SEI film formed on the surface of the N-S@HDM-1300 anode after cycling.

**29.** **Correlation between physical properties and electrochemical properties for N-S@HDM**


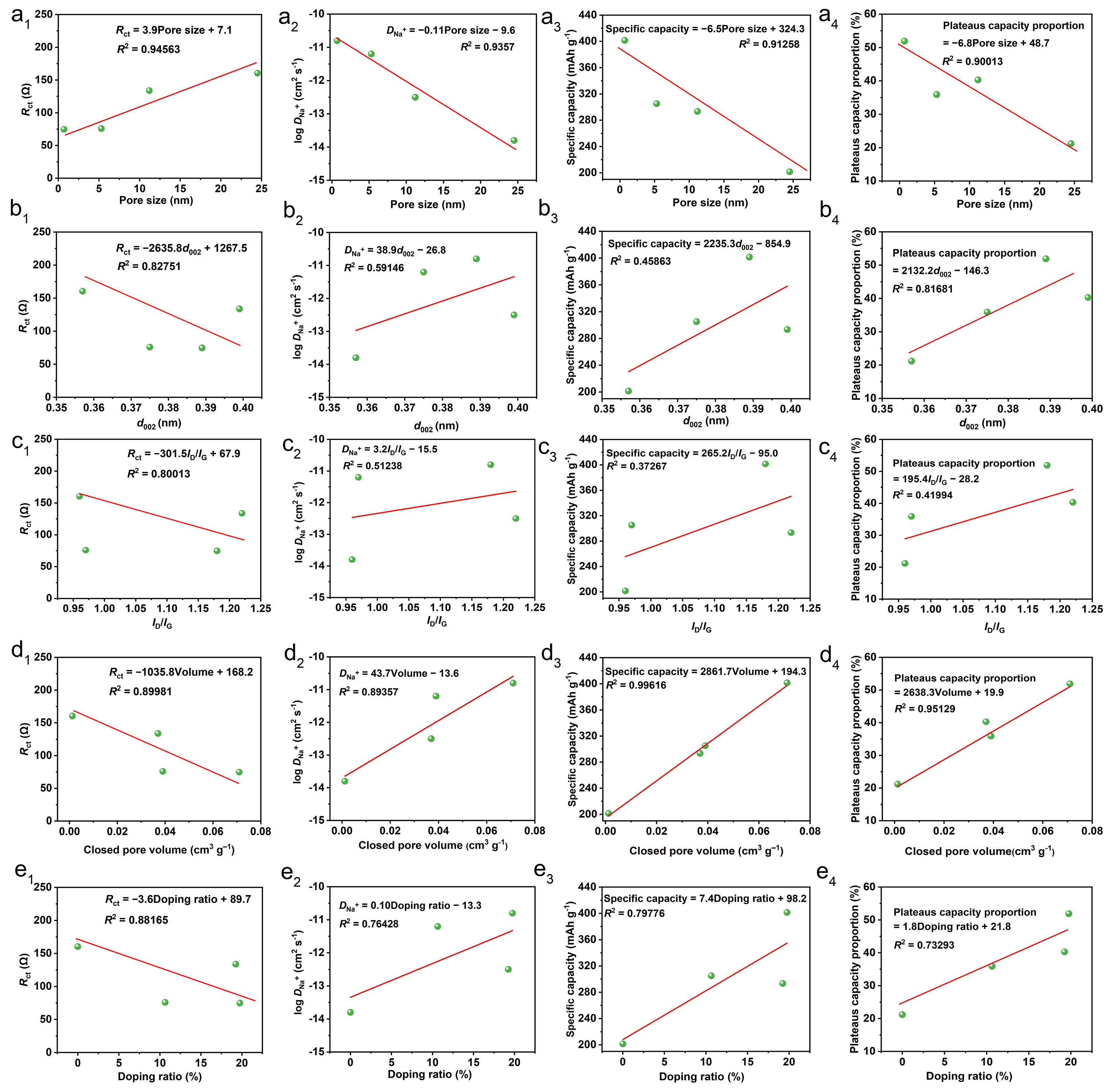


**Figure S19**. Correlation analysis of different physical properties (pore size, *d*_002_, *I*_D_/*I*_G_, closed pore volume, doping ratio) with *R*_ct_ (a_1_, b_1_, c_1_, d_1_, and e_1_), *D*_Na_^+^ (a_2_, b_2_, c_2_, d_2_, and e_2_), specific capacity (a_3_, b_3_, c_3_, d_3_, and e_3_), and plateau capacity proportion (a_4_, b_4_, c_4_, d_4_, and e_4_).

**30.** **Contents of C, O, S, and N elements and their bonds in the DFT model**

**Table S15.** Proportions of C, O, N, and S elements in N-S@HDM-1300 determined by EDX analysis and the corresponding atom numbers in the DFT structure model.

|  | C | O | N | S |
| --- | --- | --- | --- | --- |
| Proportions determined by EDX analysis | 72.68% | 7.57% | 11.22% | 8.53% |
| Number of atoms in the DFT structure model | 146 | 10 | 20 | 13 |

**Table S16.** Proportions of the chemical bonds in N-S@HDM-1300 determined by XPS and the corresponding numbers of chemical bonds in the DFT structure model.

| XPS spectra | Chemical bonds | Proportions of integral areas determined by XPS | Proportions of chemical bonds considering the elemental contents | Number of chemical bonds in the DFT structure model |
| --- | --- | --- | --- | --- |
| O 1s | C‒O‒C | 23.53% | 0.93% | 1 |
|  | C=O | 23.42% | 0.89% | 1 |
|  | COOR | 39.04% | 1.39% | 2 |
|  | O-H | 14.01% | 0.40% | — |
| N 1s | N‒O | 15.02% | 0.62% | 1 |
|  | N‒5 | 31.09% | 3.31% | 6 |
|  | N‒6 | 30.77% | 4.05% | 7 |
|  | N‒G | 23.12% | 3.24% | 6 |
| S 2p | C‒S | 54.62% | 4.57% | 7 |
|  | S‒O | 45.38% | 3.96% | 6 |
|  | C‒C/C=C | — | 72.68% | 146 |

**31. Computational details on the density functional theory (DFT) analysis**

First-principle calculation was performed by Vienna Ab Initio Simulation Package (VASP) [29, 30]. The Perdew-Burke-Ernzerhof (PBE) version of generalized gradient approximation (GGA) was performed to account for the electron-electron exchange and correlation interactions and was used throughout [31].

A plane-wave basis set was selected to expand the wave functions and the cutoff kinetic energy was aimed at 450 eV. Supercells with 4×4 graphene unit cells have 50 C atoms and 3 N or 2 S atoms in the graphene layers. All positions of the atoms were allowed to relax during the geometry optimization process. Atomic structures were optimized during the residual forces below 0.05 eV Å^‒1^. Brillouin-zone integrations were conducted using a Monkhorst–Pack k-point grid of special points (a separation of 0.06 Å^‒1^). The convergence criterion for the electronic self-consistent field (SCF) loop was attained at 2×10^-6^ eV/atom. We construct a surface model by a vacuum layer in the depth of 20 Å to separate the surface slab from its periodic duplicates. The DFT-D3 method of Grimme was adopted to correct the long-range van der Waals interaction in all the calculations [32].

In this study, two-dimensional carbon with 50 carbon atoms is used as the basic carbon material. Different doping models were established to determine the optimized geometry by modeling graphene with various N-S doping sites (Figures S20a-d). It can be found that type c is more energetically stable because its formation energy (‒8.77 eV) is 4.86, 3.49, and 3.37 eV lower than that of a, b, and d. Therefore, we select the c configuration for the following studies, including adsorption energy, differential charge density, work function, and density of states.


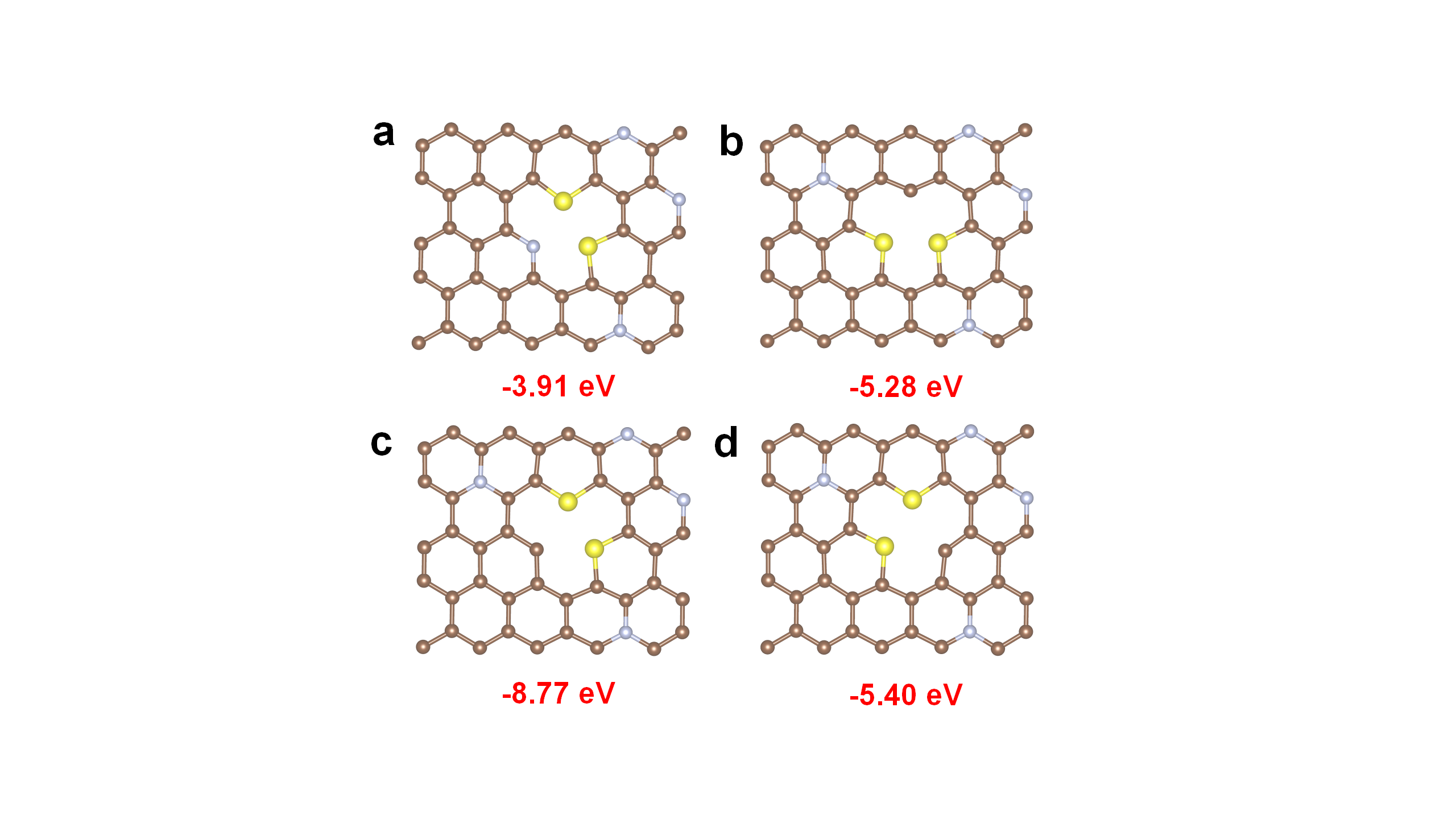


**Figure S20.** Various configurations of N and S doping sites within a representative unit of N-S@HDM-1300 (yellow, blue, and brown balls represent S, N, and C atoms, respectively).

**Supporting references**

1. Holzwarth, U. & Gibson, N. The scherrer equation versus the ‘debye-scherrer equation’. *Nature Nanotech.* **6**, 534–534 (2011).

2. Pope, C. G. X-ray diffraction and the bragg equation. *J. Chem. Educ.* **74**, 129 (1997).

3. Saurel, D. *et al.* A SAXS outlook on disordered carbonaceous materials for electrochemical energy storage. *Energy Storage Mater.* **21**, 162–173 (2019).

4. Hou, W. *et al.* Fractal dimension revealed from SAXS as a descriptor of structural disorder in hard carbon anodes of sodium ion battery. *Chin. Chem. Lett.* 111124 (2025).

5. Kim, M. *et al.* Ultra-stable sodium ion storage of biomass porous carbon derived from sugarcane. *Chem. Eng. J.* **445**, 136344 (2022).

6. Meng, Q. *et al.* Hard carbon anodes for sodium-ion batteries: dependence of the microstructure and performance on the molecular structure of lignin. *J. Power Sources.* **581**, 233475 (2023).

7. Yan, L. *et al.* In-situ graphene-coated carbon microsphere as high initial coulombic efficiency anode for superior Na/K-ion full cell. *Chem. Eng. J.* **432**, 133257 (2022).

8. Wang, Y. *et al.* Releasing free radicals in precursor triggers the formation of closed pores in hard carbon for sodium‐ion batteries. *Adv. Mater.* **36**, 2401249 (2024).

9. Tang, Y. *et al.* Electrochemical behavior of the biomass hard carbon derived from waste corncob as a sodium-ion battery anode. *Energy Fuels.* **38**, 7389–7398 (2024).

10. Liu, H. *et al.* High-performance sodium ion capacitor constructed by well-matched dual carbon electrodes from a single biomass. *ACS Sustainable Chem. Eng*. **25**, 17-37 (2019).

11. Ren, X. *et al.* Lath-shaped biomass derived hard carbon as anode materials with super rate capability for sodium-ion batteries. *J. Electroanal. Chem.* **841**, 63–72 (2019).

12. Li, Y. *et al.* Amorphous monodispersed hard carbon micro-spherules derived from biomass as a high performance negative electrode material for sodium-ion batteries. *J. Mater. Chem. A.* **3**, 71–77 (2015).

13. Zhang, N. *et al.* High capacity hard carbon derived from lotus stem as anode for sodium ion batteries. *J. Power Sources.* **378**, 331–337 (2018).

14. Li, Y. *et al.* Regulating pore structure of hierarchical porous waste cork‐derived hard carbon anode for enhanced Na storage performance. *Adv. Energy Mater.* **9**, 1902852 (2019).

15. Li, Y., Hu, Y., Titirici, M., Chen, L. & Huang, X. Hard carbon microtubes made from renewable cotton as high‐performance anode material for sodium‐ion batteries. *Adv. Energy Mater.* **6**, 1600659 (2016).

16. Yu, Z.-E. *et al.* Hard carbon micro-nano tubes derived from kapok fiber as anode materials for sodium-ion batteries and the sodium-ion storage mechanism. *Chem. Commun.* **56**, 778–781 (2020).

17. Wang, J. *et al.* Facile hydrothermal treatment route of reed straw-derived hard carbon for high performance sodium ion battery. *Electrochim. Acta.* **291**, 188–196 (2018).

18. Hu, H.-Y. *et al.* A stable biomass‐derived hard carbon anode for high‐performance sodium‐ion full battery. *Energy Technol.* **9**, 2000730 (2021).

19. Muruganantham, R., Hsieh, T.-H., Lin, C.-H. & Liu, W.-R. Bio-oil derived hierarchical porous hard carbon from rubber wood sawdust via a template fabrication process as highly stable anode for sodium-ion batteries. *Mater. Today Energy.* **14**, 100346 (2019).

20. Pei, L. *et al.* Hard carbon derived from waste tea biomass as high-performance anode material for sodium-ion batteries. *Ionics.* **26**, 5535–5542 (2020).

21. Li, C. *et al.* Heteroatom-doped hierarchically porous carbons derived from cucumber stem as high-performance anodes for sodium-ion batteries. *J. Mater. Sci.* **54**, 5641–5657 (2019).

22. Manas, R. P. *et al.* Blocks of molybdenum ditelluride: A high rate anode for sodium-ion battery and full cell prototype study. *Nano Energy.* **64**, 103951 (2019).

23. Feier, N. *et al.* MoSe_2_-covered N,P-doped carbon nanosheets as a long-life and high-rate anode material for sodium-ion batteries. *Adv. Funct. Mater.* **27**, 1700522 (2017).

24. Azusa, K. *et al.* MgO-template synthesis of extremely high capacity hard carbon for Na-ion battery. *Angew. Chem. Int. Ed.* **60**, 55114-5120 (2020).

25. Lu, Z. *et al.* Zinc single-atom-regulated hard carbons for high-rate and low-temperature sodium-ion batteries. *Adv. Mater.* **35**, 2211461 (2023).

26. Gu, J. *et al.* Creating rich closed nanopores in anthracite-derived soft carbon enables greatly-enhanced sodium-ion storage in the low-working-voltage region. *Chem. Eng. J.* **505**, 159331 (2025).

27. Wang, Q. *et al.* Rice husk-derived hard carbons as high-performance anode materials for sodium-ion batteries. *Carbon.* **127**, 658-666 (2018).

28. Xu, Z. *et al.* Tailoring sodium intercalation in graphite for high energy and power sodium ion batteries. *Nat. Commun.* **10**, 2598 (2019).

29. Kresse, G. & Furthmüller, J. Efficiency of ab-initio total energy calculations for metals and semiconductors using a plane-wave basis set. *Comp. Mater. Sci.* **6**, 15–50 (1996).

30. Kresse, G. & Joubert, D. From ultrasoft pseudopotentials to the projector augmented-wave method. *Phys. Rev. B.* **59**, 1758–1775 (1999).

31. Perdew, J. P., Burke, K. & Ernzerhof, M. Generalized gradient approximation made simple. *Phys. Rev. Lett.* **77**, 3865–3868 (1996).

32. Grimme, S., Antony, J., Ehrlich, S. & Krieg, H. A consistent and accurate *ab initio* parametrization of density functional dispersion correction (DFT-D) for the 94 elements H-Pu. *J. Chem. Phys.* **132**, 154104 (2010).
